# Causal Inference of Adverse Drug Events in Pulmonary Arterial Hypertension: A Pharmacovigilance Study

**DOI:** 10.3390/ph18081084

**Published:** 2025-07-22

**Authors:** Hongmei Li, Xiaojun He, Cui Chen, Qiao Ni, Linghao Ni, Jiawei Zhou, Bin Peng

**Affiliations:** Department of Health Statistics, College of Public Health, Chongqing Medical University, Chongqing 401331, China

**Keywords:** pulmonary arterial hypertension, adverse drug events, Bayesian causal network, pharmacovigilance

## Abstract

**Objective:** Pulmonary arterial hypertension (PAH) is a progressive and life-threatening disease. Adverse events (AEs) related to its drug treatment seriously damaged the patient’s health. This study aims to clarify the causal relationship between PAH drugs and these AEs by combining pharmacovigilance signal detection with the Bayesian causal network model. **Methods:** Patient data were obtained from the U.S. Food and Drug Administration (FDA) Adverse Event Reporting System (FAERS), covering reports from 2013 to 2023. In accordance with standard pharmacovigilance methodologies, disproportionality analysis was performed to detect signals. Target drugs were selected based on the following criteria: number of reports (a) ≥ 3, proportional reporting ratio (PRR) ≥ 2, and chi-square (χ^2^) ≥ 4. Bayesian causal network models were then constructed to estimate causal relationships. The do-calculus and adjustment formula were applied to calculate the causal effects between drugs and AEs. **Results:** Signal detection revealed that Ambrisentan, Bosentan, and Iloprost were associated with serious AEs, including death, dyspnea, pneumonia, and edema. For Ambrisentan, the top-ranked adverse drug events (ADEs) based on average causal effect (ACE) were peripheral swelling (ACE = 0.032) and anemia (ACE = 0.021). For Iloprost, the most prominent ADE was hyperthyroidism (ACE = 0.048). **Conclusions:** This study quantifies causal drug–event relationships in PAH using Bayesian causal networks. The findings offer valuable evidence regarding the clinical safety of PAH medications, thereby improving patient health outcomes.

## 1. Introduction

Pulmonary arterial hypertension (PAH) is a progressive and fatal condition, clinically defined as a mean pulmonary arterial pressure (mPAP) ≥ 20 mm Hg [1]. Its clinical features include progressively elevated pulmonary vascular pressure and increased vascular resistance resulting from pathological remodeling of small pulmonary arteries [2]. Without timely intervention, patients with PAH may undergo progressive right ventricular remodeling, ultimately leading to irreversible cardiac decompensation and hemodynamic collapse, thereby significantly increasing all-cause mortality [3]. In 2021, mainland China and India reported the highest numbers of disability-adjusted life years (DALYs), deaths, and prevalence of PAH, accounting for 38.3%, 45.1%, and 33.8% of global totals, respectively [4].

Endothelin receptor antagonists (ERAs) are considered first-line therapy for PAH [5]. ERAs exert their therapeutic effects by competitively inhibiting the binding of endothelin-1 (ET-1) to its G protein-coupled receptors, thereby disrupting the signal transduction pathways of the endothelin system. Prostacyclin analogs (PGI_2_/PPA) function by activating the IP receptor–mediated second messenger system, initiating the cAMP–PKA signaling cascade, which regulates vascular smooth muscle tone and inhibits cellular proliferation [6]. Ambrisentan and Bosentan are commonly prescribed ERAs. Bosentan exerts its antagonistic effect by competitively inhibiting the dimerization of ETA and ETB receptors. Ambrisentan is a non-sulfonamide, highly selective ETA receptor antagonist. Iloprost, a synthetic analog of prostacyclin PGI_2_, may be administered via inhalation or intravenous infusion [7]. Common adverse reactions associated with these agents include peripheral edema, nasal congestion, anemia, and cough [8], all of which may adversely affect patients’ quality of life. Developing precise monitoring strategies for adverse drug events (ADEs) holds significant clinical value, as such strategies may enhance treatment adherence and reduce hospital readmission rates.

Common ADEs associated with ERAs include liver function abnormalities, gastrointestinal disturbances, peripheral edema, and anemia [9]. Among these, hepatotoxicity is relatively rare but presents a significant threat to patient safety. In Western countries, drug-induced liver injury (DILI) has emerged as one of the leading causes of acute liver failure [10]. A post-marketing surveillance study of Ambrisentan conducted in South Korea reported an overall adverse event (AE) incidence of 52.22% and an adverse drug reaction (ADR) rate of 10.92%, with the most frequently reported ADRs being edema and headache [11]. In a randomized controlled trial (RCT) of Treprostinil, monitoring data indicated that the incidence of headache exceeded 70%, and jaw pain was reported in more than 20% of patients [12]. A high proportion of serious ADEs has also been reported for Bosentan, Ambrisentan, and Macitentan, with the most frequently cited preferred terms (PTs) including death, dyspnea, and infectious pneumonia [13].

Most existing pharmacovigilance studies focus on the associations between drugs and ADEs [14,15]. However, correlation does not equate to causation. Therefore, the objective of this study is to determine the causal relationships and effects between PAH treatments—Ambrisentan, Bosentan, and Iloprost—and their associated ADEs, thereby providing a robust scientific foundation for clinical decision-making.

## 2. Results

### 2.1. Basic Characteristics of Report

Based on the inclusion and exclusion criteria, a total of 14,224,681 reports and 41,757,311 AEs from the FAERS database between Q1 2013 and Q4 2023 were included in this study. According to the clinical indications for cardiovascular treatment, three commonly used medications for PAH were selected: Ambrisentan, Bosentan, and Iloprost. Demographic characteristics are presented in Table 1. A total of 70,083 reports were identified for Ambrisentan, involving 4128 AEs; 16,608 reports for Bosentan, involving 3595 events; and 3632 reports for Iloprost, involving 1573 events. Among the Ambrisentan reports, 52,328 (74.66%) involved female patients, and 16,839 (24.03%) involved male patients. Patients aged 18–64 years accounted for 31,361 reports (44.75%), while those aged 65–85 years accounted for 26,877 reports (38.35%). The most frequently reported outcomes for Ambrisentan were hospitalization (26,569 reports, 28.57%) and other serious AEs (29,361 reports, 31.57%). For Bosentan, 3680 deaths (18.51%), 7428 hospitalizations (37.36%), and 3292 other serious AEs (16.56%) were recorded. For Iloprost, 1840 deaths (38.48%), 1674 hospitalizations (35.01%), and 924 other serious AEs (19.32%) were reported.

**Figure 1:**
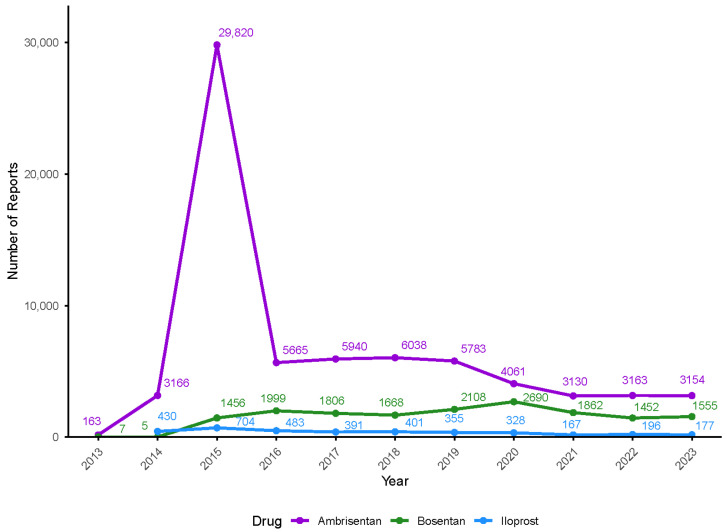
The number of adverse drug event (ADE) reports from 2013 to 2023.

The annual trend in reporting from 2013 to 2023 is illustrated in Figure 1. The peak in Ambrisentan-related reports in 2015 may be linked to the FDA label update and increased clinical use following the AMBITION trial, which supported combination therapy with tadalafil. This has likely led to greater reporting activity.

### 2.2. Drug Risk Signals

According to the criteria for DPA (number of reports ≥ 3, PRR ≥ 2, and χ^2^ ≥ 4), signals of ADEs were detected. For Ambrisentan, the five most frequently reported ADEs were dyspnea, death, pneumonia, headache, and dizziness. For Bosentan, the top five ADEs included death, dyspnea, product dose omission, pneumonia, and hospitalization. For Iloprost, the leading ADEs were death, dyspnea, hospitalization, cough, and PAH. All three drugs were associated with serious ADEs, including death, dyspnea, pneumonia, and edema (Table 2). The complete results can be found in Supplementary Materials Tables S1–S3.

### 2.3. The Causal Relationship and Effects of ADEs

**Figure 2:**
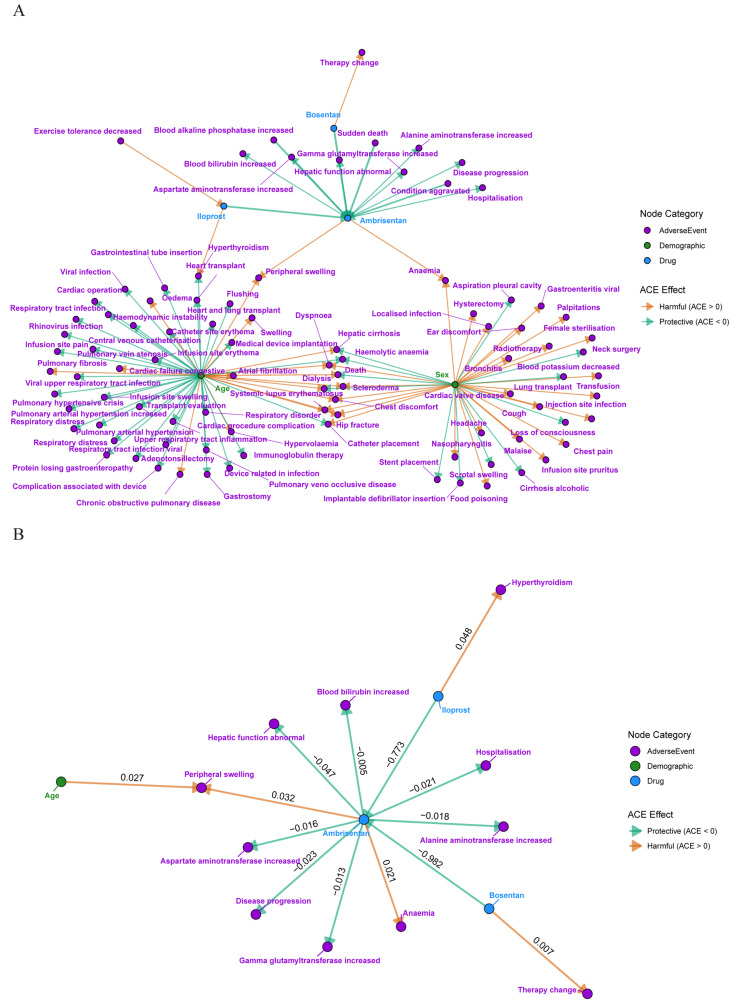
Causal diagram of ADEs in the treatment of PAH (**A**) and Causal diagram of local ADEs (**B**).

Based on the Bayesian causal graph model, a causal diagram was developed to illustrate the relationships between PAH treatments and ADEs (Figure 2). In the diagram, blue nodes represent drugs ($D_{i}$), Purple nodes represent ADEs ($A_{i}$), and green nodes represent demographic variables ($e_{i}$). Directed edges between nodes indicate causal relationships, with the arrow pointing toward the effect variables. By applying *do*-intervention and adjustment formulas to control for confounding factors (conditioning set), the direct causal effects between $D_{i}$ and ADEs ($A_{i}$) were calculated. The causal relationships with the highest ACE values included Iloprost → Hyperthyroidism (0.048), Ambrisentan → Peripheral swelling (0.032), and Ambrisentan → Anemia (0.021), as shown in Table 3. These ACE values were subsequently visualized in the causal graph. The color of the edge represents the positive or negative effect of the ACE value.

## 3. Discussion

In this study, DPA and a Bayesian causal graph model were used to build a Bayesian causal network of ADEs associated with Ambrisentan, Bosentan, and Iloprost. The causal effects between each drug and its corresponding ADEs were quantitatively estimated. Signal detection results revealed that all three drugs were associated with serious ADEs, including death, dyspnea, pneumonia, and edema. The ADEs with the strongest estimated causal effects were Hyperthyroidism, Peripheral swelling, and Anemia. This causal network illustrates the drug–ADE pathways and supports the development of personalized pharmacotherapy strategies in clinical settings.

Several clinical trials have been conducted to investigate the efficacy and safety of drugs used in the treatment of PAH. One study reported that combination therapy with Ambrisentan and Tadalafil led to a higher incidence of ADEs [16]. In comparisons of treatment effectiveness in the first treatment of the patient, parenteral prostacyclin analogs and oral Treprostinil were more likely to cause treatment discontinuation due to ADEs [17,18]. A multicenter clinical trial found that the most common drug-related ADEs were edema (38.7%) and headache (22.5%) [19]. Another trial reported that treatment with Bosentan resulted in significant liver function abnormalities, suggesting a potential hepatotoxic effect [20]. While Ambrisentan and Bosentan show comparable efficacy in PAH treatment, Ambrisentan has shown better hepatic safety, with a significantly lower incidence of liver function abnormalities [21]. However, due to limitations in clinical trials, such as the need for large sample sizes and inconsistent patient compliance, the use of spontaneous reporting systems for drug safety surveillance has received growing attention for detecting ADE signals.

Common spontaneous reporting systems include VigiBase and the FAERS. A real-world drug safety study based on FAERS identified strong pharmacovigilance signals for ADEs, including infections, cor pulmonale, right ventricular failure, fluid retention, and PAH [14]. Patients with PAH are inherently at elevated risk for right heart failure, and the use of Letairis may exacerbate cardiac complications, a factor that necessitates caution during both diagnosis and therapeutic planning [22]. An assessment of FAERS reports related to Riociguat reported frequently occurring ADEs such as headache, dizziness, hypotension, nausea, falls, and loss of consciousness [23]. FAERS data further indicated serious ADEs linked to Orenitram, such as pulmonary edema, ascites, and ventricular fibrillation [24]. The signal detection results of the present study were consistent with prior studies, each demonstrating positive signals for death, dyspnea, pneumonia, and edema.

Causal discovery is a core concept in biomedical informatics and plays a critical role in improving disease diagnosis, treatment, and prognosis. In epidemiology and clinical medicine, probabilistic causal inference methods are widely employed to investigate relationships among environmental exposures, diseases, and AEs associated with medications. Among these methods, Bayesian networks (BNs) have been extensively applied in healthcare and biomedical research [25]. To enhance the understanding of potential causal links between pharmaceuticals and ADEs, several studies have combined pharmacovigilance signal detection with causal inference approaches. For instance, based on data from the FAERS database, one study identified associations involving 63 antipsychotic agents and 5121 reports of seizure-related ADEs. Mendelian randomization (MR) analysis further revealed potential causal links between 14 drug target genes and epilepsy or its subtypes [26]. Another study identified 78 drugs associated with urinary retention and found that genetic markers related to amlodipine were significantly associated with an elevated risk of urinary retention, as confirmed by MR analysis [27]. Causal discovery based on BNs aims to identify the causal graph that best fits the data, enabling the construction of Bayesian causal models directly from real-world data. For example, one study employed BN techniques to construct protein signaling networks by incorporating causal dependencies between variables, thereby improving the structural accuracy of the model [28]. Building on pharmacovigilance signal detection results, this study applied a Bayesian causal graph model to construct a causal network of ADEs. The highest-ranking ADEs based on their ACE values were Hyperthyroidism, Peripheral swelling, and Anemia. These findings provide valuable insights for optimizing medication strategies in the treatment of PAH.

This study has several limitations. The quality of reports in the FAERS database varies significantly, including considerable missing demographic and clinical data. Additionally, most reports originate from the United States, which may introduce geographic bias and limit the generalizability of the results. Although known confounders were adjusted for during causal effect estimation, the absence of detailed clinical information may still result in residual bias. Future research should integrate multi-source data, including clinical records and genomic information, to better control for confounding variables and more accurately investigate the causal relationships between PAH therapies and ADEs.

## 4. Materials and Methods

### 4.1. Data Sources

The data for this study were sourced from the U.S. Food and Drug Administration (FDA) Adverse Event Reporting System (FAERS) database. The dataset comprises four components: the demographic information table (DEMO), the drug usage table (DRUG), the AE report table (REAC), and the patient outcome table (OUTC).

### 4.2. Data Processing Procedure

Inclusion criteria: (1) AE reports dated between 1 January 2013 and 31 December 2023; (2) Reports involving the use of Ambrisentan, Bosentan, or Iloprost; (3) AEs identified using preferred terms (PTs) from version 27 of the Medical Dictionary for Regulatory Activities (MedDRA). Exclusion criterion: (1) Duplicate reports.

Age was standardized in units of years. Reports with missing sex and age information were excluded from the analysis. Age, originally a continuous variable, was subsequently converted into a categorical variable.

**Figure 3:**
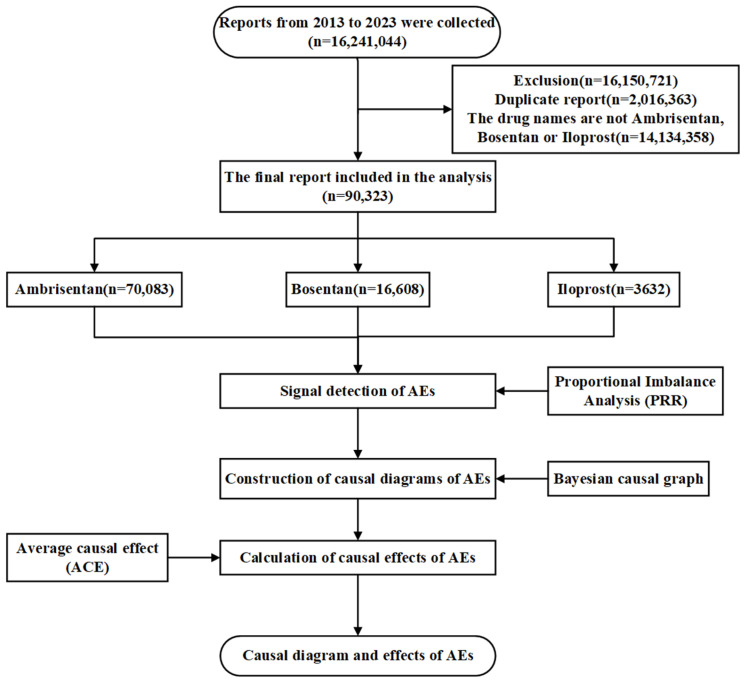
Research and design flowchart.

The DEMO, DRUG, and REAC were merged using the unique report identifier “Primaryid”. Variables retained for analysis included: Primaryid, sex, age, drug, AE, and patient outcome. Subsequently, the data structure was modified by converting the drug variable into three binary indicators representing Ambrisentan, Bosentan, and Iloprost (0 = not used, 1 = used). ADEs were encoded at the Preferred Term (PT) level following MedDRA terminology. Each PT-level ADE was converted into a separate binary variable, where 0 indicated the absence and 1 indicated the presence of the specific event. The overall data processing workflow is illustrated in Figure 3.

### 4.3. Drug Signal Detection

According to standard pharmacovigilance methodology, all individuals in the database who used drugs other than the target drugs and experienced ADEs were designated as the control group, forming a case/non-case study design. A signal was considered present when the proportion of individuals reporting a specific ADE after taking a target drug was significantly higher than the proportion of individuals taking other drugs and reporting the same ADE. Signal detection was conducted based on the following criteria: number of reports for the target drug (a) ≥ 3, proportional reporting ratio (PRR) ≥ 2, and chi-square (χ^2^) ≥ 4.

(1) $$PRR=\frac{a/(a+b)}{c/(c+d)}$$

(2) $$x^{2}=\frac{\left(a+b+c+d\right){\left(ad-bc\right)}^{2}}{\left(a+b\right)\left(a+c\right)\left(c+d\right)\left(b+d\right)}$$

### 4.4. Construction of Bayesian Causal Graph Model

The basic structure of the graphical model is as follows:

(1) Node X: Represents a random variable. Each node corresponds to a specific drug (Dᵢ), adverse drug event (ADE, Aᵢ), or confounder (eᵢ).
(2) Directed edge E: Represents a causal relationship. The direction of the edge flows from the drug (Dᵢ) to the ADE (Aᵢ). For example, a directed edge from drug D_1_ to ADE A_1_ indicates that D_1_ is a potential cause of A_1_.
(3) Three fundamental graphical structures: chain structure, fork structure, and V-structure.

D-Separation Criterion (Independence Between Node Sets X):

A path L between nodes is said to be blocked by a conditioning set of nodes Z (i.e., variables D and A are conditionally independent given Z) if and only if:

(1) L contains a chain structure D → e → A or a fork structure D ← e → A, where the intermediate node e is in the conditioning set Z;
(2) L contains a V-structure A → e ← C, where the intermediate node e is not in Z, and none of its descendants are in Z.

Mutual information is an information theoretical measure used to assess the dependency between two vertices (i.e., variables) in a network. It quantifies how much information about one variable (e.g., ADE ($A_{i}$)) can be obtained from knowledge of another variable (e.g., ($D_{i}$)). In other words, mutual information measures the reduction in uncertainty of ADE ($A_{i}$) given ($D_{i}$). The mutual information $MI(D_{i},A_{i})$ is defined as the Kullback–Leibler divergence (relative entropy) between the joint distribution $p(D_{i},A_{i})$ and the product of the marginal distributions $p\left(D_{i}\right)p(A_{i})$.

(3) $$MI\left(D_{i},A_{i}\right)=\sum_{D_{i},A_{i}}p(D_{i},A_{i})log\frac{p\left(D_{i},A_{i}\right)}{p\left(D_{i}\right)p\left(A_{i}\right)}$$

A smaller value of mutual information $MI(D_{i},A_{i})$ indicates that $A_{i}$ contains less information about $D_{i}$, suggesting a higher likelihood of independence between $D_{i}$ and $A_{i}$ increases. Mutual information is symmetric, meaning that, MI ($D_{i}$, $A_{i}$) = MI ($A_{i}$, $D_{i}$). For a given finite set of variables Z ($D_{i}$,$A_{i}$,$e_{i}$), the conditional mutual information between $D_{i}$ and $A_{i}$ given Z can also be estimated.

(4) $$MI\left(D_{i},A_{i}|Z\right)=\sum_{D_{i},A_{i},Z}p(D_{i},A_{i},Z)log\frac{p\left(D_{i},A_{i}|Z\right)}{p\left(D_{i}|Z\right)p\left(A_{i}|Z\right)}$$

Similarly, a smaller value of conditional mutual information $MI\left(D_{i},A_{i}|Z\right)$ suggests a higher likelihood that $D_{i}$ and $A_{i}$ are conditionally independent given Z. In this study, under the null hypothesis of conditional independence, the test statistic $2nMI\left(D_{i},A_{i}|Z\right)$ approximately follows a chi-square distribution. A *p*-value greater than 0.05 indicates that the variables are considered conditionally independent.

Given the prior knowledge from signal detection, specifically, the directed edges from known $D_{i}$ to $A_{i}$, the Bayesian causal graph was constructed through the following steps: (1) Skeleton Identification: A fully connected undirected graph was initially constructed based on prior constraints, incorporating all variables in the dataset, including $D_{i}$, $A_{i}$ and $e_{i}$ such as age, sex. The conditional independence test is based on the conditional mutual information test, and the edges with *p*-values greater than 0.05 are removed to reflect the conditional independence relationship, forming the initial skeleton. (2) V-Structure Identification: V-structures were identified within the skeleton using the d-separation criterion and appropriate conditioning sets Z. (3) Edge Orientation: The direction of the remaining edges is determined by the orientation rules (R1–R4) [29].

To eliminate the influence of $e_{i}$, an intervention was applied on the causal graph using the do-intervention, which involves removing all incoming edges to the $D_{i}$. Based on the adjustment formula and excluding the confounding factor e, the average causal effect (ACE) of the $D_{i}$ and the $A_{i}$ was subsequently estimated.

(5) $$ACE=P\left(A=1|do\left(D=1\right)\right)-P\left(A=1|do\left(D=0\right)\right)$$

(6) $$P(A=a|do(D=d))=\sum_{e}P(A=a|D=d,e=e)P(e=e)$$

### 4.5. Statistical Analysis

All data analyses in this study were conducted using R software (version 4.3.3). Categorical variables in the demographic dataset were summarized as counts and percentages (N (%)). Disproportionality analysis (DPA) was performed to detect ADE signals based on the number of reports for the target drug (a), PRR, and chi-square statistics. A Bayesian causal graph model was used to construct the drug–ADE causal network. The ACE between drugs and ADEs was estimated using the do-intervention and adjustment formula. The clinical validity of the final network was assessed by comparing the identified drug–ADE relationships with known associations reported in regulatory databases and the literature. All statistical tests were two-sided, and a *p*-value less than 0.05 was considered statistically significant.

## 5. Conclusions

In this study, a DPA combined with a Bayesian causal network model was used to construct causal networks of ADEs associated with Ambrisentan, Bosentan, and Iloprost. The causal effects between these drugs and ADEs were quantitatively estimated. The top ADEs ranked by causal effect included Hyperthyroidism, Peripheral swelling, and Anemia. This model provides a scientifically grounded and precise reference for clinicians in developing individualized treatment strategies, thereby contributing to improved patient outcomes and overall health status.

## Figures and Tables

**Table 1:** Demographic characteristics of drug reports for pulmonary arterial hypertension (PAH).

Characteristics	Ambrisentan	Bosentan	Iloprost
	(*n* = 70,083)	(*n* = 16,608)	(*n* = 3632)
Sex (%)			
Female	52,328 (74.66)	9562 (57.57)	2385 (65.67)
Male	16,839 (24.03)	3399 (20.47)	1074 (29.57)
Missing	916 (1.31)	3647 (21.96)	173 (4.76)
Age (years, %)			
≤17	1492 (2.13)	1623 (9.77)	71 (1.95)
18~64	31,361 (44.75)	3956 (23.82)	1517 (41.77)
65~85	26,877 (38.35)	4113 (24.77)	1221 (33.62)
≥86	1802 (2.57)	509 (3.06)	58 (1.60)
Missing	8551 (12.20)	6407 (38.58)	765 (21.06)
Occupation (%)			
Consumer	42,081 (60.04)	3857 (23.22)	234 (6.44)
Health-professional	21,703(30.97)	10,765(64.82)	2829(77.89)
Other	4665 (6.66)	1946 (11.72)	564 (15.53)
Missing	1634 (2.33)	40 (0.24)	5 (0.14)
Outcome (%)	*n* = 93004	*n* = 19881	*n* = 4782
DE	8788 (9.45)	3680 (18.51)	1840 (38.48)
LT	354 (0.38)	200 (1.01)	64 (1.34)
HO	26,569 (28.57)	7428 (37.36)	1674 (35.01)
DS	350 (0.38)	176 (0.89)	32 (0.67)
CA	19 (0.02)	18 (0.09)	2 (0.04)
RI	26 (0.03)	3 (0.01)	0(0.00)
OT	29,361 (31.57)	3292 (16.56)	924 (19.32)
Missing	27,537 (29.60)	5084 (25.57)	246 (5.14)

DE: Death, LT: Life-Threatening, HO: Hospitalization—Initial or Prolonged, DS: Disability, CA: Congenital Anomaly, RI: Required Intervention to Prevent Permanent Impairment/Damage, OT: Other Serious (Important Medical Event).

**Table 2:** The proportion reporting ratio of ADEs in the treatment of PAH.

Drug	ADE	a	b	c	d	PRR	$x^{2}$
Ambrisentan							
	Dyspnea	10,591	165,188	369,466	41,212,066	6.78	51,209.29
	Death	6538	169,241	595,980	40,985,552	2.60	6433.15
	Pneumonia	4113	171,666	227,925	41,353,607	4.27	10,168.89
	Headache	3930	171,849	428,461	41,153,071	2.17	2481.63
	Dizziness	3177	172,602	324,768	41,256,764	2.31	2366.34
	Fluid retention	3097	172,682	33,975	41,547,557	21.56	55,707.13
	Malaise	2823	172,956	317,678	41,263,854	2.10	1629.35
	Peripheral swelling	2624	173,155	124,156	41,457,376	5.00	8246.90
	Edema peripheral	2292	173,487	59,088	41,522,444	9.18	16,097.13
	Edema	2180	173,599	31,858	41,549,674	16.19	29,097.02
Bosentan							
	Death	2142	56,143	600,376	41,098,650	2.55	2044.94
	Dyspnea	2097	56,188	377,960	41,321,066	3.97	4674.91
	Product dose omission issue	1231	57,054	374,305	41,324,721	2.35	963.12
	Pneumonia	930	57,355	231,108	41,467,918	2.88	1142.25
	Hospitalization	761	57,524	109,489	41,589,537	4.97	2404.86
	Cough	613	57,672	194,764	41,504,262	2.25	427.22
	Pulmonary arterial hypertension	606	57,679	10,949	41,688,077	39.60	21,609.63
	Condition aggravated	570	57,715	203,730	41,495,296	2.00	286.31
	Chest pain	478	57,807	112,108	41,586,918	3.05	657.78
	Fluid retention	463	57,822	36,609	41,662,417	9.05	3276.00
Iloprost							
	Death	1194	11,878	601,324	41,142,915	6.34	5439.16
	Dyspnea	455	12,617	379,602	41,364,637	3.83	958.05
	Hospitalization	208	12,864	110,042	41,634,197	6.04	874.64
	Cough	194	12,878	195,183	41,549,056	3.17	289.96
	Pulmonary arterial hypertension	368	25,776	24,544	83,463,934	47.88	16,647.08
	Pneumonia	151	12,921	231,887	41,512,352	2.08	85.03
	Product use issue	140	12,932	150,565	41,593,674	2.97	183.35
	Inappropriate schedule of product administration	139	12,933	184,355	41,559,884	2.41	114.83
	Fluid retention	129	12,943	36,943	41,707,296	11.15	1188.95
	Chest pain	122	12,950	112,464	41,631,775	3.46	214.19

Only the first ten examples are shown.

**Table 3:** The causal effects of ADEs in the treatment of PAH.

Drug	ADE	Conditional Set	ACE
Iloprost	Hyperthyroidism	None	0.048
Ambrisentan	Peripheral swelling	Age, Iloprost	0.032
Ambrisentan	Anemia	None	0.021
Bosentan	Therapy change	None	0.007
Ambrisentan	Blood bilirubin increased	None	−0.005
Ambrisentan	Gamma-glutamyltransferase increased	None	−0.013
Ambrisentan	Aspartate aminotransferase increased	None	−0.016
Ambrisentan	Alanine aminotransferase increased	None	−0.018
Ambrisentan	Hospitalization	None	−0.021
Ambrisentan	Disease progression	None	−0.023
Ambrisentan	Hepatic function abnormal	None	−0.047
Iloprost	Ambrisentan	None	−0.773
Bosentan	Ambrisentan	None	−0.982

## Data Availability

The original data presented in the study are openly available in the FAERS database.

## Supplementary Materials

The following supporting information can be downloaded at: https://www.mdpi.com/article/10.3390/ph18081084/s1, Table S1: The proportion reporting ratio of ADEs related to Ambrisentan. Table S2: The proportion reporting ratio of the ADEs related to Bosentan. Table S3: The proportion reporting ratio of the ADEs related to Iloprost.

## References

[1] Maron B.A. (2023). Revised Definition of Pulmonary Hypertension and Approach to Management: A Clinical Primer. J. Am. Heart Assoc..

[2] Humbert M., Kovacs G., Hoeper M.M., Badagliacca R., Berger R.M.F., Brida M., Carlsen J., Coats A.J.S., Escribano-Subias P., Ferrari P. (2022). 2022 ESC/ERS Guidelines for the diagnosis and treatment of pulmonary hypertension Developed by the task force for the diagnosis and treatment of pulmonary hypertension of the European Society of Cardiology (ESC) and the European Respiratory Society (ERS). Endorsed by the International Society for Heart and Lung Transplantation (ISHLT) and the European Reference Network on rare respiratory diseases (ERN-LUNG). Eur. Heart J..

[3] Mocumbi A., Humbert M., Saxena A., Jing Z.C., Sliwa K., Thienemann F., Archer S.L., Stewart S. (2024). Pulmonary hypertension. Nat. Rev. Dis. Primers.

[4] Yang Y., Zeng Z., Yang Q., Wang H., Zhang H., Yan W., Wang P., Wang C., Su Z., Thangaraju P. (2025). The Challenge in Burden of Pulmonary Arterial Hypertension: A Perspective From the Global Burden of Disease Study. MedComm.

[5] Klinger J.R., Elliot C.G., Levine D.J., Bossone E., Duvall L., Fagan K., Frantsve-Hawley J., Kawut S.M., Ryan J.J., Rosenzweig E.B. (2021). Therapy for Pulmonary Arterial Hypertension in Adults: Update of the CHEST Guideline and Expert Panel Report (vol 155, pg 565, 2019). Chest.

[6] Chin K.M., Gaine S.P., Gerges C., Jing Z.C., Mathai S.C., Tamura Y., McLaughlin V.V., Sitbon O. (2024). Treatment algorithm for pulmonary arterial hypertension. Eur. Respir. J..

[7] Zolty R. (2023). Advances in the discovery of drugs that treat pulmonary arterial hypertension. Expert. Opin. Drug Dis..

[8] Tamargo J., Agewall S., Ambrosio G., Borghi C., Cerbai E., Dan G.A., Drexel H., Ferdinandy P., Grove E.L., Klingenberg R. (2025). New pharmacological agents and novel cardiovascular pharmacotherapy strategies in 2024. Eur. Heart J.-Card. Pha.

[9] Zhang Y.J., Wang N., Gu Z.C., Wei A.H., Cheng A.N., Fang S.S., Du H.L., Wang L.Z., Zhang G.Q. (2019). A network meta-analysis for safety of endothelin receptor antagonists in pulmonary arterial hypertension. Cardiovasc. Diagn. The.

[10] Weber S., Gerbes A.L. (2022). Challenges and Future of Drug-Induced Liver Injury Research-Laboratory Tests. Int. J. Mol. Sci..

[11] Jeon K., Yoo S.B., Lee Y.H., Lee E.B., Kim H.K., Chang H.J., Chang S.A. (2023). Safety and effectiveness of ambrisentan in real clinical practice in pulmonary arterial hypertension: Results from the Korean post-marketing surveillance. Pharmacoepidem Dr. S.

[12] Barnes H., Yeoh H.L., Fothergill T., Burns A., Humbert M., Williams T. (2019). Prostacyclin for pulmonary arterial hypertension. Cochrane Database Syst. Rev..

[13] Tang F.J., Ma Q.H., Liu Y.H., Yang X.J. (2025). Risk of respiratory, thoracic, and mediastinal disorders associated with endothelin receptor antagonists and prostacyclin-related drugs in pulmonary hypertension: A disproportionality analysis based on FAERS. Expert. Opin. Drug Saf..

[14] Bi Y.T., Dong B. (2024). Clinical adverse events to letairis: A real-world drug safety study based on FDA Adverse Event Reporting System (FAERS). Expert. Opin. Drug Saf..

[15] Gu J.J., Guo Y.T., Wu B., He J.H. (2024). Liver injury associated with endothelin receptor antagonists: A pharmacovigilance study based on FDA adverse event reporting system data. Int. J. Clin. Pharm-Net..

[16] Fu W.H., He W.J., Li Y.X., Chen Y.X., Liang J.Y., Lei H., Fu L., Chen Y.H., Ren N., Jiang Q. (2021). Efficacy and safety of novel-targeted drugs in the treatment of pulmonary arterial hypertension: A Bayesian network meta-analysis. Drug Deliv..

[17] Petrovic M., Locatelli I. (2020). Comparative effectiveness of pulmonary arterial hypertension drugs in treatment-naive patients: A network meta-analysis. J. Comp. Effect Res..

[18] Wang P.W., Deng J.X., Zhang Q.Y., Feng H.Y., Zhang Y.H., Lu Y.Z., Han L.Z., Yang P.F., Deng Z.J. (2022). Additional Use of Prostacyclin Analogs in Patients With Pulmonary Arterial Hypertension: A Meta-Analysis. Front. Pharmacol..

[19] Preston I.R., Burger C.D., Bartolome S., Safdar Z., Krowka M., Sood N., Ford H.J., Battarjee W.F., Chakinala M.M., Gomberg-Maitland M. (2020). Ambrisentan in portopulmonary hypertension: A multicenter, open-label trial. J. Heart Lung Transpl..

[20] Chen X.W., Zhai Z.G., Huang K., Xie W.M., Wan J., Wang C. (2018). Bosentan therapy for pulmonary arterial hypertension and chronic thromboembolic pulmonary hypertension: A systemic review and meta-analysis. Clin. Respir. J..

[21] Zhao Q.H., Guo N., Chen J., Parks D., Tian Z. (2022). Comparative assessment of efficacy and safety of ambrisentan and bosentan in patients with pulmonary arterial hypertension: A meta-analysis. J. Clin. Pharm. Ther..

[22] Weatherald J., Hemnes A.R., Maron B.A., Mielniczuk L.M., Gerges C., Price L.C., Hoeper M.M., Humbert M. (2024). Phenotypes in pulmonary hypertension. Eur. Respir. J..

[23] Wang L.L., Mao Z.Y., Zheng P.D., Zi G.S., Zhang F.Q., Zhu X.Y., Chen L.X., Liu H.G., Zhou L., Wei S. (2025). Assessment of Riociguat-related adverse events: A disproportionality analysis utilizing the FDA adverse event reporting system database. Expert. Opin. Drug Saf..

[24] Chai S.J., Xu H.M., Xu G.C., Cai C.M. (2024). ORENITRAM’s decadal journey: Unveiling safety profiles and adverse event through a real-world pharmacovigilance study of FAERS events. Expert. Opin. Drug Saf..

[25] Ahangaran M., Jahed-Motlagh M.R., Minaei-Bidgoli B. (2020). A novel method for predicting the progression rate of ALS disease based on automatic generation of probabilistic causal chains. Artif. Intell. Med..

[26] Yin Z.Q., Zhan Z., Qiu Y.J., Wang M.H., Li J.L., Song B.Y., Chen Z.Q., Wu J., Wang Z. (2025). Exploring the Relationship Between Antipsychotic Drug Target Genes and Epilepsy: Evidence From Food and Drug Administration Adverse Event Reporting System Database and Mendelian Randomization. Brain Behav..

[27] Zhang W., Yang F., Li W.C., Ma Y.P., Ma Z.F., Wang X., Hu C.Y. (2024). Drugs Associated with Urinary Retention Adverse Reactions: A Joint Analysis of FDA Adverse Event Reporting System and Mendelian Randomization. Urology.

[28] Wei X.H., Zhang Y.L., Wang C. (2022). Bayesian Network Structure Learning Method Based on Causal Direction Graph for Protein Signaling Networks. Entropy.

[29] Kalisch M., Bühlmann P. (2007). Estimating high-dimensional directed acyclic graphs with the PC-algorithm. J. Mach. Learn. Res..

