# Supplementary material for: Causal Inference of Adverse Drug Events in Pulmonary Arterial Hypertension: A Pharmacovigilance Study
Source: Pharmaceuticals (Basel). 2025 Jul 22;18(8):1084. doi: 10.3390/ph18081084 (PMC12388902; doi:10.3390/ph18081084)
Supplement: Supplementary file 1 [file pharmaceuticals-18-01084-s001.zip › pharmaceuticals-3726101-supplementary.pdf]

**Table S1:** The proportion reporting ratio of ADEs related to Ambrisentan.

| PT | a | b | c | d | PRR | χ2 |
|---|---|---|---|---|---|---|
| Dyspnoea | 10591 | 165188 | 369466 | 41212066 | 6.78 | 51209.29 |
| Death | 6538 | 169241 | 595980 | 40985552 | 2.6 | 6433.15 |
| Pneumonia | 4113 | 171666 | 227925 | 41353607 | 4.27 | 10168.89 |
| Headache | 3930 | 171849 | 428461 | 41153071 | 2.17 | 2481.63 |
| Dizziness | 3177 | 172602 | 324768 | 41256764 | 2.31 | 2366.34 |
| Fluid retention | 3097 | 172682 | 33975 | 41547557 | 21.56 | 55707.13 |
| Malaise | 2823 | 172956 | 317678 | 41263854 | 2.1 | 1629.35 |
| Peripheral swelling | 2624 | 173155 | 124156 | 41457376 | 5 | 8246.9 |
| Oedema peripheral | 2292 | 173487 | 59088 | 41522444 | 9.18 | 16097.13 |
| Oedema | 2180 | 173599 | 31858 | 41549674 | 16.19 | 29097.02 |
| Cough | 2173 | 173606 | 193204 | 41388328 | 2.66 | 2237.61 |
| Nasal congestion | 2168 | 173611 | 38565 | 41542967 | 13.3 | 23368.34 |
| Unevaluable event | 2166 | 173613 | 53137 | 41528395 | 9.64 | 16142.77 |
| Chest pain | 1884 | 173895 | 110702 | 41470830 | 4.03 | 4224.39 |
| Syncope | 1728 | 174051 | 62017 | 41519515 | 6.59 | 7985.83 |
| Hypotension | 1604 | 174175 | 129962 | 41451570 | 2.92 | 2006.05 |
| Swelling | 1489 | 174290 | 79425 | 41502107 | 4.43 | 3895.78 |
| Pulmonary arterial hypertension | 1489 | 174290 | 10066 | 41571466 | 34.99 | 42843.92 |
| Nasopharyngitis | 1375 | 174404 | 131098 | 41450434 | 2.48 | 1206.89 |
| Oxygen saturation decreased | 1263 | 174516 | 35753 | 41545779 | 8.36 | 7907.32 |
| Pulmonary hypertension | 1180 | 174599 | 12177 | 41569355 | 22.92 | 22562.38 |
| Cardiac failure congestive | 1164 | 174615 | 49276 | 41532256 | 5.59 | 4288.65 |
| Anaemia | 1121 | 174658 | 126074 | 41455458 | 2.1 | 645.07 |
| Palpitations | 1034 | 174745 | 74148 | 41507384 | 3.3 | 1636.56 |
| Dyspnoea exertional | 1002 | 174777 | 24706 | 41556826 | 9.59 | 7417.53 |
| Flushing | 870 | 174909 | 59476 | 41522056 | 3.46 | 1502.1 |
| Influenza | 807 | 174972 | 77306 | 41504226 | 2.47 | 699.63 |
| Cardiac failure | 795 | 174984 | 51536 | 41529996 | 3.65 | 1507.58 |
| Chest discomfort | 703 | 175076 | 65163 | 41516369 | 2.55 | 657.51 |
| Hypervolaemia | 699 | 175080 | 14140 | 41567392 | 11.69 | 6516.16 |
| Loss of consciousness | 687 | 175092 | 76006 | 41505526 | 2.14 | 413.26 |
| Cardiac disorder | 664 | 175115 | 59790 | 41521742 | 2.63 | 662.74 |

| | | | | | | |
|---|---|---|---|---|---|---|
| Atrial fibrillation | 650 | 175129 | 63024 | 41518508 | 2.44 | 547.44 |
| Epistaxis | 635 | 175144 | 51934 | 41529598 | 2.89 | 777.69 |
| Therapy interrupted | 635 | 175144 | 38016 | 41543516 | 3.95 | 1378.06 |
| Bronchitis | 617 | 175162 | 52265 | 41529267 | 2.79 | 702.58 |
| Sinusitis | 612 | 175167 | 72016 | 41509516 | 2.01 | 308.64 |
| Respiratory failure | 585 | 175194 | 44165 | 41537367 | 3.13 | 839.51 |
| Pain in jaw | 575 | 175204 | 19206 | 41562326 | 7.08 | 2917.5 |
| Heart rate increased | 546 | 175233 | 61926 | 41519606 | 2.09 | 306.34 |
| Chronic obstructive pulmonary disease | 541 | 175238 | 34290 | 41547242 | 3.73 | 1066.15 |
| Intentional dose omission | 525 | 175254 | 13630 | 41567902 | 9.11 | 3651.86 |
| Lung disorder | 523 | 175256 | 31546 | 41549986 | 3.92 | 1120.78 |
| Catheterisation cardiac | 488 | 175291 | 2587 | 41578945 | 44.62 | 17509.49 |
| Right ventricular failure | 471 | 175308 | 4463 | 41577069 | 24.96 | 9802.09 |
| Dialysis | 466 | 175313 | 7541 | 41573991 | 14.62 | 5568.91 |
| Hypoxia | 459 | 175320 | 21327 | 41560205 | 5.09 | 1477.98 |
| Cellulitis | 385 | 175394 | 34264 | 41547268 | 2.66 | 394.08 |
| Device related infection | 360 | 175419 | 11375 | 41570157 | 7.49 | 1961.74 |
| Lung transplant | 329 | 175450 | 2536 | 41578996 | 30.69 | 8364.81 |
| Productive cough | 325 | 175454 | 32665 | 41548867 | 2.35 | 250.71 |
| Acute respiratory failure | 314 | 175465 | 12428 | 41569104 | 5.98 | 1269.54 |
| Pulmonary oedema | 313 | 175466 | 28086 | 41553446 | 2.64 | 314.59 |
| Scleroderma | 304 | 175475 | 2208 | 41579324 | 32.57 | 8177.12 |
| Presyncope | 295 | 175484 | 16040 | 41565492 | 4.35 | 747.78 |
| Infusion site pain | 268 | 175511 | 8972 | 41572560 | 7.07 | 1355.46 |
| Haemoptysis | 267 | 175512 | 18399 | 41563133 | 3.43 | 453.96 |
| Pericardial effusion | 266 | 175513 | 14227 | 41567305 | 4.42 | 691.93 |
| Systemic lupus erythematosus | 261 | 175518 | 22102 | 41559430 | 2.79 | 297.18 |
| Sinus disorder | 254 | 175525 | 14026 | 41567506 | 4.28 | 628.23 |
| Throat irritation | 249 | 175530 | 29447 | 41552085 | 2 | 123.6 |
| Sinus congestion | 246 | 175533 | 8872 | 41572660 | 6.56 | 1128.03 |
| Liver function test abnormal | 243 | 175536 | 15245 | 41566287 | 3.77 | 487.12 |
| Hip fracture | 233 | 175546 | 21069 | 41560463 | 2.62 | 230.18 |
| Head injury | 227 | 175552 | 20515 | 41561017 | 2.62 | 224.53 |
| Viral infection | 227 | 175552 | 21290 | 41560242 | 2.52 | 206.45 |

| | | | | | | |
|---|---|---|---|---|---|---|
| Blood potassium decreased | 207 | 175572 | 18852 | 41562680 | 2.6 | 201.25 |
| Central venous catheterisation | 202 | 175577 | 2114 | 41579418 | 22.6 | 3807.32 |
| Localised infection | 200 | 175579 | 17693 | 41563839 | 2.67 | 207.34 |
| Oxygen consumption increased | 194 | 175585 | 1646 | 41579886 | 27.88 | 4497.92 |
| Transfusion | 187 | 175592 | 8310 | 41573222 | 5.32 | 642.25 |
| Clostridium difficile infection | 181 | 175598 | 17182 | 41564350 | 2.49 | 160.06 |
| Respiratory disorder | 171 | 175608 | 19141 | 41562391 | 2.11 | 99.45 |
| Ascites | 170 | 175609 | 18718 | 41562814 | 2.15 | 103.47 |
| Exercise tolerance decreased | 165 | 175614 | 3804 | 41577728 | 10.26 | 1321.89 |
| Gout | 161 | 175618 | 12115 | 41569417 | 3.14 | 232.33 |
| Respiratory tract infection | 160 | 175619 | 17512 | 41564020 | 2.16 | 98.98 |
| Cardiac operation | 159 | 175620 | 5190 | 41576342 | 7.25 | 830.88 |
| Gastroenteritis viral | 152 | 175627 | 12254 | 41569278 | 2.93 | 191.49 |
| Respiratory distress | 150 | 175629 | 16527 | 41565005 | 2.15 | 91.12 |
| Infusion site erythema | 147 | 175632 | 5472 | 41576060 | 6.35 | 646.03 |
| Cardiac pacemaker insertion | 142 | 175637 | 3547 | 41577985 | 9.47 | 1034.45 |
| Internal haemorrhage | 138 | 175641 | 9795 | 41571737 | 3.33 | 222.25 |
| Pulmonary fibrosis | 136 | 175643 | 11351 | 41570181 | 2.83 | 159.58 |
| Complication associated with device | 132 | 175647 | 15338 | 41566194 | 2.04 | 69 |
| Pulmonary thrombosis | 132 | 175647 | 7525 | 41574007 | 4.15 | 310.17 |
| Heart rate irregular | 126 | 175653 | 14236 | 41567296 | 2.09 | 71.38 |
| Hepatic cirrhosis | 125 | 175654 | 11569 | 41569963 | 2.56 | 117.16 |
| Oxygen therapy | 122 | 175657 | 1508 | 41580024 | 19.14 | 1940.29 |
| Blood iron decreased | 116 | 175663 | 8122 | 41573410 | 3.38 | 191.55 |
| Sleep apnoea syndrome | 116 | 175663 | 13511 | 41568021 | 2.03 | 60.21 |
| Generalised oedema | 114 | 175665 | 6896 | 41574636 | 3.91 | 242.98 |
| Seasonal allergy | 103 | 175676 | 10102 | 41571430 | 2.41 | 84.29 |
| Upper respiratory tract congestion | 101 | 175678 | 1842 | 41579690 | 12.97 | 1057.88 |
| Terminal state | 101 | 175678 | 4993 | 41576539 | 4.79 | 296.45 |
| Dizziness postural | 98 | 175681 | 6125 | 41575407 | 3.78 | 197.68 |
| Pneumothorax | 96 | 175683 | 10604 | 41570928 | 2.14 | 57.91 |
| Liver transplant | 94 | 175685 | 1949 | 41579583 | 11.41 | 851.66 |
| Pulmonary congestion | 94 | 175685 | 7640 | 41573892 | 2.91 | 116.47 |
| Oxygen saturation abnormal | 92 | 175687 | 1853 | 41579679 | 11.74 | 861.62 |

| | | | | | | |
|---|---|---|---|---|---|---|
| Pulmonary arterial pressure increased | 91 | 175688 | 1097 | 41580435 | 19.62 | 1485.19 |
| Infusion site swelling | 91 | 175688 | 4207 | 41577325 | 5.12 | 295.07 |
| Vascular device infection | 90 | 175689 | 3181 | 41578351 | 6.69 | 423.85 |
| Respiration abnormal | 89 | 175690 | 4749 | 41576783 | 4.43 | 232.31 |
| Therapy change | 89 | 175690 | 6654 | 41574878 | 3.16 | 130.01 |
| Stent placement | 88 | 175691 | 4895 | 41576637 | 4.25 | 215.09 |
| Hysterectomy | 87 | 175692 | 5330 | 41576202 | 3.86 | 181.52 |
| Mechanical ventilation | 86 | 175693 | 1026 | 41580506 | 19.83 | 1418.69 |
| Atrial flutter | 85 | 175694 | 4956 | 41576576 | 4.06 | 192.53 |
| Cyanosis | 81 | 175698 | 8473 | 41573059 | 2.26 | 56.47 |
| Gallbladder disorder | 81 | 175698 | 9345 | 41572187 | 2.05 | 43.22 |
| Ear discomfort | 79 | 175700 | 6883 | 41574649 | 2.72 | 84.63 |
| Secretion discharge | 77 | 175702 | 8820 | 41572712 | 2.07 | 41.95 |
| Catheter placement | 75 | 175704 | 1275 | 41580257 | 13.92 | 849.1 |
| Catheter site erythema | 74 | 175705 | 1461 | 41580071 | 11.98 | 708.93 |
| Intra-abdominal fluid collection | 74 | 175705 | 1582 | 41579950 | 11.07 | 647.26 |
| Endotracheal intubation | 73 | 175706 | 1685 | 41579847 | 10.25 | 583.98 |
| Lower respiratory tract congestion | 72 | 175707 | 1317 | 41580215 | 12.93 | 751.64 |
| Transplant evaluation | 71 | 175708 | 379 | 41581153 | 44.32 | 2531.73 |
| Catheter site pain | 71 | 175708 | 1697 | 41579835 | 9.9 | 545.09 |
| Nasal dryness | 70 | 175709 | 4289 | 41577243 | 3.86 | 146.02 |
| Dyspnoea at rest | 70 | 175709 | 2071 | 41579461 | 8 | 414.46 |
| Localised oedema | 69 | 175710 | 2990 | 41578542 | 5.46 | 245.66 |
| Infusion site infection | 69 | 175710 | 1472 | 41580060 | 11.09 | 605 |
| Respiratory syncytial virus infection | 67 | 175712 | 5070 | 41576462 | 3.13 | 95.63 |
| Iron deficiency anaemia | 66 | 175713 | 5953 | 41575579 | 2.62 | 65.54 |
| Cardiomegaly | 62 | 175717 | 6324 | 41575208 | 2.32 | 46.08 |
| Cardiac failure acute | 59 | 175720 | 4304 | 41577228 | 3.24 | 90.29 |
| Left ventricular failure | 58 | 175721 | 1956 | 41579576 | 7.01 | 290.51 |
| Cholecystectomy | 58 | 175721 | 6374 | 41575158 | 2.15 | 35.47 |
| Knee operation | 56 | 175723 | 6268 | 41575264 | 2.11 | 32.56 |
| Catheter site infection | 54 | 175725 | 2632 | 41578900 | 4.85 | 161.9 |
| Chemotherapy | 53 | 175726 | 2287 | 41579245 | 5.48 | 189.83 |
| Heart valve replacement | 53 | 175726 | 912 | 41580620 | 13.75 | 592.06 |

| | | | | | | |
|---|---|---|---|---|---|---|
| Catheter site haemorrhage | 52 | 175727 | 1578 | 41579954 | 7.8 | 298.21 |
| Carbon dioxide increased | 52 | 175727 | 847 | 41580685 | 14.52 | 616.91 |
| Ulcer haemorrhage | 50 | 175729 | 4483 | 41577049 | 2.64 | 50.31 |
| Sarcoidosis | 50 | 175729 | 2942 | 41578590 | 4.02 | 111.56 |
| Polyuria | 49 | 175730 | 5309 | 41576223 | 2.18 | 31.14 |
| Cardiac valve disease | 49 | 175730 | 3330 | 41578202 | 3.48 | 85.39 |
| Sickle cell anaemia | 48 | 175731 | 397 | 41581135 | 28.6 | 1140.64 |
| Orthopnoea | 47 | 175732 | 1909 | 41579623 | 5.82 | 183.3 |
| Catheter site pruritus | 46 | 175733 | 712 | 41580820 | 15.28 | 576.78 |
| Ammonia increased | 46 | 175733 | 3196 | 41578336 | 3.4 | 77.03 |
| Middle ear effusion | 45 | 175734 | 1476 | 41580056 | 7.21 | 233.67 |
| Paranasal sinus discomfort | 45 | 175734 | 2835 | 41578697 | 3.75 | 89.54 |
| Viral upper respiratory tract infection | 45 | 175734 | 4013 | 41577519 | 2.65 | 45.82 |
| Nocturnal dyspnoea | 44 | 175735 | 796 | 41580736 | 13.08 | 465.01 |
| Paranasal sinus hypersecretion | 43 | 175736 | 2640 | 41578892 | 3.85 | 89.39 |
| Heart rate abnormal | 43 | 175736 | 3784 | 41577748 | 2.69 | 45.08 |
| Limb operation | 42 | 175737 | 3191 | 41578341 | 3.11 | 59.48 |
| Brain natriuretic peptide increased | 42 | 175737 | 1843 | 41579689 | 5.39 | 146.87 |
| Tracheostomy | 42 | 175737 | 854 | 41580678 | 11.63 | 389.11 |
| Infusion site pruritus | 41 | 175738 | 2096 | 41579436 | 4.63 | 114.35 |
| Cardiac flutter | 41 | 175738 | 4137 | 41577395 | 2.34 | 31.3 |
| Infusion site haemorrhage | 41 | 175738 | 2292 | 41579240 | 4.23 | 99.41 |
| Colonoscopy | 40 | 175739 | 1444 | 41580088 | 6.55 | 183.15 |
| Injection site infection | 40 | 175739 | 2051 | 41579481 | 4.61 | 111.05 |
| Catheter site discharge | 39 | 175740 | 741 | 41580791 | 12.45 | 390.17 |
| Food poisoning | 39 | 175740 | 3477 | 41578055 | 2.65 | 39.74 |
| Rhinovirus infection | 39 | 175740 | 2473 | 41579059 | 3.73 | 76.74 |
| Sinus headache | 38 | 175741 | 3649 | 41577883 | 2.46 | 32.7 |
| Positive airway pressure therapy | 38 | 175741 | 371 | 41581161 | 24.23 | 767.67 |
| Heart transplant | 37 | 175742 | 550 | 41580982 | 15.91 | 484.54 |
| Thrombosis in device | 37 | 175742 | 1962 | 41579570 | 4.46 | 97.52 |
| Renal transplant | 37 | 175742 | 2135 | 41579397 | 4.1 | 85.24 |
| Cardiac ablation | 36 | 175743 | 1214 | 41580318 | 7.01 | 180.32 |
| Chronic respiratory failure | 36 | 175743 | 734 | 41580798 | 11.6 | 332.48 |

| | | | | | | |
|---|---|---|---|---|---|---|
| Ear congestion | 35 | 175744 | 1822 | 41579710 | 4.54 | 94.93 |
| Painful respiration | 35 | 175744 | 1586 | 41579946 | 5.22 | 116.84 |
| Cor pulmonale | 35 | 175744 | 513 | 41581019 | 16.14 | 465.3 |
| Gastric infection | 35 | 175744 | 2845 | 41578687 | 2.91 | 43.35 |
| Transplant | 35 | 175744 | 1615 | 41579917 | 5.13 | 113.8 |
| Non-cardiac chest pain | 33 | 175746 | 1779 | 41579753 | 4.39 | 84.76 |
| Eye operation | 33 | 175746 | 2822 | 41578710 | 2.77 | 36.79 |
| Infusion site discharge | 33 | 175746 | 858 | 41580674 | 9.1 | 229.07 |
| Wheelchair user | 32 | 175747 | 2999 | 41578533 | 2.52 | 29.14 |
| Life support | 32 | 175747 | 289 | 41581243 | 26.19 | 698.11 |
| Raynaud's phenomenon | 32 | 175747 | 3155 | 41578377 | 2.4 | 25.85 |
| Gallbladder operation | 32 | 175747 | 2356 | 41579176 | 3.21 | 48.12 |
| Haemorrhoidal haemorrhage | 32 | 175747 | 3502 | 41578030 | 2.16 | 19.79 |
| Pulmonary veno-occlusive disease | 31 | 175748 | 440 | 41581092 | 16.67 | 426.48 |
| Pneumonia viral | 30 | 175749 | 2153 | 41579379 | 3.3 | 47.33 |
| Deafness unilateral | 29 | 175750 | 3300 | 41578232 | 2.08 | 16.1 |
| Idiopathic pulmonary fibrosis | 29 | 175750 | 2798 | 41578734 | 2.45 | 24.68 |
| Apparent death | 29 | 175750 | 2902 | 41578630 | 2.36 | 22.6 |
| Breath sounds abnormal | 28 | 175751 | 3291 | 41578241 | 2.01 | 14.15 |
| Enteritis infectious | 28 | 175751 | 1224 | 41580308 | 5.41 | 98.44 |
| Aortic stenosis | 28 | 175751 | 2051 | 41579481 | 3.23 | 42.52 |
| Pulmonary pain | 28 | 175751 | 2231 | 41579301 | 2.97 | 36.11 |
| Atypical pneumonia | 27 | 175752 | 2255 | 41579277 | 2.83 | 31.63 |
| Iron deficiency | 26 | 175753 | 2992 | 41578540 | 2.06 | 13.97 |
| Respiratory tract infection viral | 26 | 175753 | 1484 | 41580048 | 4.14 | 60.96 |
| Blood potassium abnormal | 26 | 175753 | 1783 | 41579749 | 3.45 | 44.58 |
| Gastric bypass | 26 | 175753 | 1231 | 41580301 | 5 | 81.39 |
| Oxygen consumption | 25 | 175754 | 107 | 41581425 | 55.27 | 1079.9 |
| Ventilation perfusion mismatch | 25 | 175754 | 172 | 41581360 | 34.38 | 707.48 |
| Infusion site rash | 24 | 175755 | 910 | 41580622 | 6.24 | 102.87 |
| Leg amputation | 24 | 175755 | 2598 | 41578934 | 2.19 | 15.29 |
| Catheter site swelling | 24 | 175755 | 646 | 41580886 | 8.79 | 159.72 |
| Gastrointestinal tube insertion | 24 | 175755 | 1466 | 41580066 | 3.87 | 50.32 |
| Paracentesis | 23 | 175756 | 447 | 41581085 | 12.17 | 224.3 |

| | | | | | | |
|---|---|---|---|---|---|---|
| Cardioversion | 23 | 175756 | 943 | 41580589 | 5.77 | 88.53 |
| Dizziness exertional | 22 | 175757 | 477 | 41581055 | 10.91 | 189.31 |
| Shoulder arthroplasty | 22 | 175757 | 1771 | 41579761 | 2.94 | 27.79 |
| Walking distance test abnormal | 22 | 175757 | 291 | 41581241 | 17.88 | 326.03 |
| Nasal oedema | 22 | 175757 | 1011 | 41580521 | 5.15 | 71.96 |
| Biopsy lung | 22 | 175757 | 253 | 41581279 | 20.57 | 376.84 |
| Catheter site inflammation | 21 | 175758 | 413 | 41581119 | 12.03 | 202.07 |
| Echocardiogram abnormal | 21 | 175758 | 681 | 41580851 | 7.29 | 110.66 |
| Infusion site reaction | 20 | 175759 | 1448 | 41580084 | 3.27 | 31.04 |
| Pruritus allergic | 20 | 175759 | 875 | 41580657 | 5.41 | 70.23 |
| Lung operation | 20 | 175759 | 583 | 41580949 | 8.12 | 120.63 |
| Cardiac discomfort | 20 | 175759 | 1451 | 41580081 | 3.26 | 30.92 |
| Diastolic dysfunction | 20 | 175759 | 1792 | 41579740 | 2.64 | 20.15 |
| Large intestinal haemorrhage | 20 | 175759 | 1489 | 41580043 | 3.18 | 29.45 |
| Brain operation | 20 | 175759 | 1490 | 41580042 | 3.18 | 29.41 |
| Eye oedema | 19 | 175760 | 1529 | 41580003 | 2.94 | 24.02 |
| Dysentery | 19 | 175760 | 1404 | 41580128 | 3.2 | 28.38 |
| Chest injury | 19 | 175760 | 1476 | 41580056 | 3.05 | 25.77 |
| Female sterilisation | 19 | 175760 | 353 | 41581179 | 12.73 | 194.92 |
| Vascular graft | 19 | 175760 | 1838 | 41579694 | 2.45 | 16.07 |
| Vasodilatation | 18 | 175761 | 1619 | 41579913 | 2.63 | 17.99 |
| Injection site discharge | 18 | 175761 | 1857 | 41579675 | 2.29 | 13 |
| Cardiac pacemaker replacement | 18 | 175761 | 331 | 41581201 | 12.86 | 186.8 |
| Mitral valve disease | 18 | 175761 | 992 | 41580540 | 4.29 | 44.65 |
| Organ failure | 18 | 175761 | 1867 | 41579665 | 2.28 | 12.82 |
| Cholecystitis infective | 18 | 175761 | 1783 | 41579749 | 2.39 | 14.38 |
| Nasal disorder | 17 | 175762 | 1938 | 41579594 | 2.08 | 9.39 |
| Heart valve incompetence | 17 | 175762 | 1942 | 41579590 | 2.07 | 9.33 |
| Sinus operation | 17 | 175762 | 1619 | 41579913 | 2.48 | 14.91 |
| Catheter management | 17 | 175762 | 847 | 41580685 | 4.75 | 49.31 |
| Catheterisation cardiac abnormal | 17 | 175762 | 119 | 41581413 | 33.79 | 473.37 |
| Small intestinal haemorrhage | 17 | 175762 | 1345 | 41580187 | 2.99 | 22.23 |
| Catheter removal | 17 | 175762 | 261 | 41581271 | 15.41 | 215.03 |
| Administration site pain | 17 | 175762 | 752 | 41580780 | 5.35 | 58.76 |

| | | | | | | |
|---|---|---|---|---|---|---|
| Sickle cell disease | 17 | 175762 | 396 | 41581136 | 10.16 | 134.54 |
| Crest syndrome | 16 | 175763 | 159 | 41581373 | 23.8 | 317.58 |
| Infusion site warmth | 16 | 175763 | 831 | 41580701 | 4.55 | 43.55 |
| Menopause | 16 | 175763 | 1569 | 41579963 | 2.41 | 13.1 |
| Finger amputation | 16 | 175763 | 442 | 41581090 | 8.56 | 103.15 |
| Systemic scleroderma | 16 | 175763 | 565 | 41580967 | 6.7 | 75.44 |
| Hiv infection | 16 | 175763 | 1669 | 41579863 | 2.27 | 11.23 |
| Connective tissue disorder | 16 | 175763 | 914 | 41580618 | 4.14 | 37.47 |
| Hypercapnia | 16 | 175763 | 1809 | 41579723 | 2.09 | 9.04 |
| Infusion site irritation | 15 | 175764 | 814 | 41580718 | 4.36 | 38.13 |
| Radiotherapy | 15 | 175764 | 1171 | 41580361 | 3.03 | 20.15 |
| Device infusion issue | 15 | 175764 | 1745 | 41579787 | 2.03 | 7.81 |
| Neck surgery | 15 | 175764 | 1656 | 41579876 | 2.14 | 9.06 |
| Device connection issue | 15 | 175764 | 1116 | 41580416 | 3.18 | 22.11 |
| Aortic valve replacement | 15 | 175764 | 705 | 41580827 | 5.03 | 47.47 |
| Arteriovenous malformation | 15 | 175764 | 553 | 41580979 | 6.42 | 66.78 |
| Feeding tube user | 15 | 175764 | 243 | 41581289 | 14.6 | 179.01 |
| Mineral supplementation | 14 | 175765 | 357 | 41581175 | 9.28 | 99.48 |
| Respiratory tract irritation | 14 | 175765 | 660 | 41580872 | 5.02 | 44.11 |
| Colon operation | 14 | 175765 | 711 | 41580821 | 4.66 | 39.44 |
| Aspiration pleural cavity | 14 | 175765 | 565 | 41580967 | 5.86 | 55.09 |
| Infusion site induration | 14 | 175765 | 875 | 41580657 | 3.78 | 28.24 |
| Catheter site rash | 14 | 175765 | 358 | 41581174 | 9.25 | 99.15 |
| Pulmonary hypertensive crisis | 14 | 175765 | 165 | 41581367 | 20.07 | 233.86 |
| Pulmonary arterial pressure abnormal | 14 | 175765 | 206 | 41581326 | 16.08 | 185.35 |
| Angioplasty | 14 | 175765 | 799 | 41580733 | 4.14 | 32.83 |
| Biopsy | 13 | 175766 | 574 | 41580958 | 5.36 | 45.05 |
| Infusion site vesicles | 13 | 175766 | 383 | 41581149 | 8.03 | 77.37 |
| Implantable defibrillator insertion | 13 | 175766 | 756 | 41580776 | 4.07 | 29.57 |
| Endoscopy | 13 | 175766 | 352 | 41581180 | 8.74 | 85.89 |
| Dialysis related complication | 13 | 175766 | 276 | 41581256 | 11.14 | 114.62 |
| Device alarm issue | 13 | 175766 | 1434 | 41580098 | 2.14 | 7.87 |
| Cor pulmonale acute | 13 | 175766 | 179 | 41581353 | 17.18 | 184.69 |
| Laryngeal pain | 12 | 175767 | 269 | 41581263 | 10.55 | 99.34 |

| | | | | | | |
|---|---|---|---|---|---|---|
| Heart valve operation | 12 | 175767 | 285 | 41581247 | 9.96 | 92.82 |
| Thyroidectomy | 12 | 175767 | 1148 | 41580384 | 2.47 | 10.42 |
| Catheter site related reaction | 12 | 175767 | 378 | 41581154 | 7.51 | 65.63 |
| Mitral valve replacement | 12 | 175767 | 206 | 41581326 | 13.78 | 134.4 |
| Bladder neoplasm | 12 | 175767 | 1145 | 41580387 | 2.48 | 10.48 |
| Endarterectomy | 12 | 175767 | 153 | 41581379 | 18.55 | 184.79 |
| Ear tube insertion | 11 | 175768 | 193 | 41581339 | 13.48 | 120.27 |
| Gastric dilatation | 11 | 175768 | 1202 | 41580330 | 2.16 | 6.83 |
| Therapeutic procedure | 11 | 175768 | 834 | 41580698 | 3.12 | 15.64 |
| Blood iron abnormal | 11 | 175768 | 794 | 41580738 | 3.28 | 17.17 |
| Nasal obstruction | 11 | 175768 | 1166 | 41580366 | 2.23 | 7.41 |
| Large intestine infection | 11 | 175768 | 1285 | 41580247 | 2.02 | 5.66 |
| Chronic hepatic failure | 11 | 175768 | 400 | 41581132 | 6.51 | 49.88 |
| Cardiac infection | 11 | 175768 | 529 | 41581003 | 4.92 | 33.65 |
| Sialoadenitis | 11 | 175768 | 898 | 41580634 | 2.9 | 13.51 |
| Medical procedure | 11 | 175768 | 947 | 41580585 | 2.75 | 12.09 |
| Medical device implantation | 10 | 175769 | 886 | 41580646 | 2.67 | 10.33 |
| Echocardiogram | 10 | 175769 | 82 | 41581450 | 28.85 | 239.61 |
| Allergic respiratory symptom | 10 | 175769 | 229 | 41581303 | 10.33 | 80.74 |
| Oesophageal haemorrhage | 10 | 175769 | 841 | 41580691 | 2.81 | 11.55 |
| Dyspnoea paroxysmal nocturnal | 10 | 175769 | 389 | 41581143 | 6.08 | 41.39 |
| Aortic valve disease | 10 | 175769 | 969 | 41580563 | 2.44 | 8.42 |
| Right ventricular dysfunction | 10 | 175769 | 728 | 41580804 | 3.25 | 15.36 |
| Gastrostomy | 10 | 175769 | 1084 | 41580448 | 2.18 | 6.35 |
| Rotator cuff repair | 10 | 175769 | 1148 | 41580384 | 2.06 | 5.41 |
| Catheter site extravasation | 10 | 175769 | 345 | 41581187 | 6.86 | 48.62 |
| Palliative care | 10 | 175769 | 1010 | 41580522 | 2.34 | 7.62 |
| Rectal prolapse | 10 | 175769 | 791 | 41580741 | 2.99 | 13.08 |
| Pulmonary arterial pressure decreased | 10 | 175769 | 59 | 41581473 | 40.09 | 325.95 |
| Hereditary haemorrhagic telangiectasia | 9 | 175770 | 45 | 41581487 | 47.31 | 339.99 |
| Biopsy liver | 9 | 175770 | 275 | 41581257 | 7.74 | 51.16 |
| Suture insertion | 9 | 175770 | 337 | 41581195 | 6.32 | 39.23 |
| Dialysis device insertion | 9 | 175770 | 72 | 41581460 | 29.57 | 220.83 |
| Humidity intolerance | 9 | 175770 | 336 | 41581196 | 6.34 | 39.39 |

| | | | | | | |
|---|---|---|---|---|---|---|
| Lip discolouration | 9 | 175770 | 791 | 41580741 | 2.69 | 9.46 |
| Pulmonary arterial wedge pressure increased | 9 | 175770 | 89 | 41581443 | 23.92 | 179.52 |
| Fractured coccyx | 9 | 175770 | 846 | 41580686 | 2.52 | 8.14 |
| Hepatorenal syndrome | 9 | 175770 | 1044 | 41580488 | 2.04 | 4.73 |
| Infusion | 9 | 175770 | 310 | 41581222 | 6.87 | 43.85 |
| Ovarian cyst ruptured | 9 | 175770 | 801 | 41580731 | 2.66 | 9.2 |
| Lower respiratory tract infection bacterial | 9 | 175770 | 407 | 41581125 | 5.23 | 30.13 |
| Upper respiratory tract inflammation | 9 | 175770 | 804 | 41580728 | 2.65 | 9.13 |
| Heart and lung transplant | 9 | 175770 | 89 | 41581443 | 23.92 | 179.52 |
| Arteriovenous fistula operation | 9 | 175770 | 222 | 41581310 | 9.59 | 66.55 |
| Infusion site cellulitis | 9 | 175770 | 302 | 41581230 | 7.05 | 45.37 |
| Congestive hepatopathy | 9 | 175770 | 696 | 41580836 | 3.06 | 12.31 |
| Metapneumovirus infection | 9 | 175770 | 492 | 41581040 | 4.33 | 22.61 |
| Pregnancy test positive | 9 | 175770 | 192 | 41581340 | 11.09 | 78.91 |
| Inner ear disorder | 8 | 175771 | 633 | 41580899 | 2.99 | 10.46 |
| Irregular breathing | 8 | 175771 | 458 | 41581074 | 4.13 | 18.67 |
| Wisdom teeth removal | 8 | 175771 | 897 | 41580635 | 2.11 | 4.63 |
| Scrotal swelling | 8 | 175771 | 594 | 41580938 | 3.19 | 11.84 |
| Lower respiratory tract inflammation | 8 | 175771 | 151 | 41581381 | 12.53 | 80.63 |
| Resuscitation | 8 | 175771 | 802 | 41580730 | 2.36 | 6.21 |
| Pneumonia mycoplasmal | 8 | 175771 | 459 | 41581073 | 4.12 | 18.6 |
| Gravitational oedema | 8 | 175771 | 520 | 41581012 | 3.64 | 15.08 |
| Intensive care | 8 | 175771 | 855 | 41580677 | 2.21 | 5.27 |
| Infusion site abscess | 8 | 175771 | 214 | 41581318 | 8.84 | 53.65 |
| Central venous catheter removal | 8 | 175771 | 346 | 41581186 | 5.47 | 28.56 |
| Portopulmonary hypertension | 8 | 175771 | 68 | 41581464 | 27.83 | 185.15 |
| Pneumonia respiratory syncytial viral | 8 | 175771 | 512 | 41581020 | 3.7 | 15.49 |
| Carotid artery disease | 8 | 175771 | 618 | 41580914 | 3.06 | 10.97 |
| Critical illness | 8 | 175771 | 514 | 41581018 | 3.68 | 15.39 |
| Breast conserving surgery | 8 | 175771 | 534 | 41580998 | 3.54 | 14.39 |
| Pulmonary contusion | 7 | 175772 | 198 | 41581334 | 8.36 | 43.83 |
| Nasal inflammation | 7 | 175772 | 721 | 41580811 | 2.3 | 5.08 |
| Acute right ventricular failure | 7 | 175772 | 107 | 41581425 | 15.48 | 88.96 |
| Catheter site vesicles | 7 | 175772 | 146 | 41581386 | 11.34 | 62.99 |

| | | | | | | |
|---|---|---|---|---|---|---|
| Abdominal cavity drainage | 7 | 175772 | 317 | 41581215 | 5.22 | 23.39 |
| Carbon dioxide abnormal | 7 | 175772 | 208 | 41581324 | 7.96 | 41.22 |
| Medical device change | 7 | 175772 | 349 | 41581183 | 4.74 | 20.28 |
| Cardiac output increased | 7 | 175772 | 118 | 41581414 | 14.03 | 79.98 |
| Endometrial ablation | 7 | 175772 | 273 | 41581259 | 6.07 | 28.87 |
| Gastric antral vascular ectasia | 7 | 175772 | 324 | 41581208 | 5.11 | 22.66 |
| Blood gases abnormal | 7 | 175772 | 274 | 41581258 | 6.04 | 28.73 |
| Wrist surgery | 7 | 175772 | 641 | 41580891 | 2.58 | 6.72 |
| Cardiorenal syndrome | 7 | 175772 | 445 | 41581087 | 3.72 | 13.71 |
| Gastrointestinal viral infection | 7 | 175772 | 737 | 41580795 | 2.25 | 4.8 |
| Gastrectomy | 7 | 175772 | 759 | 41580773 | 2.18 | 4.44 |
| Procedural hypotension | 7 | 175772 | 463 | 41581069 | 3.58 | 12.8 |
| Cardiac output decreased | 7 | 175772 | 510 | 41581022 | 3.25 | 10.74 |
| Catheter site cellulitis | 7 | 175772 | 152 | 41581380 | 10.89 | 60.13 |
| Gastrointestinal arteriovenous malformation | 7 | 175772 | 184 | 41581348 | 9 | 47.95 |
| Cardiac septal defect | 7 | 175772 | 478 | 41581054 | 3.46 | 12.09 |
| Right ventricular systolic pressure increased | 7 | 175772 | 177 | 41581355 | 9.36 | 50.25 |
| Oxygen saturation increased | 7 | 175772 | 247 | 41581285 | 6.7 | 33.04 |
| Acute left ventricular failure | 7 | 175772 | 474 | 41581058 | 3.49 | 12.28 |
| Primary biliary cholangitis | 7 | 175772 | 466 | 41581066 | 3.55 | 12.65 |
| Jugular vein distension | 6 | 175773 | 326 | 41581206 | 4.35 | 15.22 |
| Sleep study | 6 | 175773 | 48 | 41581484 | 29.57 | 147.22 |
| Skin graft | 6 | 175773 | 435 | 41581097 | 3.26 | 9.29 |
| Respiratory therapy | 6 | 175773 | 56 | 41581476 | 25.35 | 126.73 |
| Brain natriuretic peptide abnormal | 6 | 175773 | 164 | 41581368 | 8.65 | 39.19 |
| Biopsy kidney | 6 | 175773 | 178 | 41581354 | 7.97 | 35.4 |
| Mixed connective tissue disease | 6 | 175773 | 258 | 41581274 | 5.5 | 21.6 |
| Pulmonary artery dilatation | 6 | 175773 | 206 | 41581326 | 6.89 | 29.36 |
| Infusion site inflammation | 6 | 175773 | 360 | 41581172 | 3.94 | 12.96 |
| Ovarian mass | 6 | 175773 | 464 | 41581068 | 3.06 | 8.21 |
| Tumour excision | 6 | 175773 | 530 | 41581002 | 2.68 | 6.24 |
| Cardiac assistance device user | 6 | 175773 | 239 | 41581293 | 5.94 | 24.04 |
| Diaphragmatic paralysis | 6 | 175773 | 364 | 41581168 | 3.9 | 12.72 |
| Toe operation | 6 | 175773 | 523 | 41581009 | 2.71 | 6.42 |

| | | | | | | |
|---|---|---|---|---|---|---|
| Angiogram | 6 | 175773 | 163 | 41581369 | 8.71 | 39.48 |
| Cardiac procedure complication | 6 | 175773 | 311 | 41581221 | 4.56 | 16.38 |
| Pulmonary endarterectomy | 6 | 175773 | 127 | 41581405 | 11.18 | 53.08 |
| Atrial septal defect repair | 6 | 175773 | 126 | 41581406 | 11.26 | 53.57 |
| Protein-losing gastroenteropathy | 6 | 175773 | 302 | 41581230 | 4.7 | 17.14 |
| Life expectancy shortened | 6 | 175773 | 360 | 41581172 | 3.94 | 12.96 |
| Cor pulmonale chronic | 6 | 175773 | 121 | 41581411 | 11.73 | 56.11 |
| Tricuspid valve disease | 6 | 175773 | 231 | 41581301 | 6.14 | 25.19 |
| Intracardiac pressure increased | 6 | 175773 | 61 | 41581471 | 23.27 | 116.41 |
| Pregnancy test negative | 6 | 175773 | 49 | 41581483 | 28.97 | 144.33 |
| Manufacturing product shipping issue | 6 | 175773 | 421 | 41581111 | 3.37 | 9.87 |
| Dental pulp disorder | 5 | 175774 | 55 | 41581477 | 21.51 | 89.61 |
| Scrotal oedema | 5 | 175774 | 449 | 41581083 | 2.63 | 5.01 |
| Physiotherapy | 5 | 175774 | 299 | 41581233 | 3.96 | 10.86 |
| Infusion site pustule | 5 | 175774 | 111 | 41581421 | 10.66 | 41.86 |
| Complications of transplanted liver | 5 | 175774 | 151 | 41581381 | 7.83 | 28.85 |
| Renal surgery | 5 | 175774 | 413 | 41581119 | 2.86 | 5.99 |
| Catheter site warmth | 5 | 175774 | 98 | 41581434 | 12.07 | 48.3 |
| Infusion site urticaria | 5 | 175774 | 433 | 41581099 | 2.73 | 5.43 |
| Vascular operation | 5 | 175774 | 256 | 41581276 | 4.62 | 13.91 |
| Corneal transplant | 5 | 175774 | 434 | 41581098 | 2.73 | 5.4 |
| Swan ganz catheter placement | 5 | 175774 | 5 | 41581527 | 236.56 | 586.4 |
| Administration site swelling | 5 | 175774 | 277 | 41581255 | 4.27 | 12.3 |
| Bleeding varicose vein | 5 | 175774 | 381 | 41581151 | 3.1 | 7.04 |
| Craniotomy | 5 | 175774 | 273 | 41581259 | 4.33 | 12.59 |
| Dependence on respirator | 5 | 175774 | 267 | 41581265 | 4.43 | 13.03 |
| Administration site infection | 5 | 175774 | 64 | 41581468 | 18.48 | 76.68 |
| Lower respiratory tract infection fungal | 5 | 175774 | 297 | 41581235 | 3.98 | 10.98 |
| Pulmonary vascular disorder | 5 | 175774 | 302 | 41581230 | 3.92 | 10.68 |
| Oesophageal food impaction | 5 | 175774 | 225 | 41581307 | 5.26 | 16.86 |
| Implantable cardiac monitor insertion | 5 | 175774 | 69 | 41581463 | 17.14 | 70.87 |
| Right ventricular dilatation | 5 | 175774 | 464 | 41581068 | 2.55 | 4.66 |
| Right-to-left cardiac shunt | 5 | 175774 | 181 | 41581351 | 6.53 | 22.81 |
| Housebound | 5 | 175774 | 329 | 41581203 | 3.6 | 9.23 |

| | | | | | | |
|---|---|---|---|---|---|---|
| Hearing aid user | 5 | 175774 | 260 | 41581272 | 4.55 | 13.58 |
| Peptic ulcer perforation | 5 | 175774 | 145 | 41581387 | 8.16 | 30.35 |
| Chronic left ventricular failure | 5 | 175774 | 258 | 41581274 | 4.58 | 13.75 |
| Intra-uterine contraceptive device insertion | 4 | 175775 | 36 | 41581496 | 26.28 | 87.56 |
| Mouth breathing | 4 | 175775 | 248 | 41581284 | 3.82 | 8.18 |
| Biopsy skin | 4 | 175775 | 111 | 41581421 | 8.52 | 25.64 |
| Computerised tomogram | 4 | 175775 | 94 | 41581438 | 10.07 | 31.33 |
| Laryngeal operation | 4 | 175775 | 31 | 41581501 | 30.52 | 101.17 |
| Immunoglobulin therapy | 4 | 175775 | 46 | 41581486 | 20.57 | 68.52 |
| Vertebroplasty | 4 | 175775 | 234 | 41581298 | 4.04 | 9.01 |
| Pericardial drainage | 4 | 175775 | 157 | 41581375 | 6.03 | 16.35 |
| Exercise lack of | 4 | 175775 | 313 | 41581219 | 3.02 | 5.35 |
| Tooth impacted | 4 | 175775 | 295 | 41581237 | 3.21 | 6 |
| Infusion site scar | 4 | 175775 | 324 | 41581208 | 2.92 | 4.99 |
| Oesophagogastric fundoplasty | 4 | 175775 | 92 | 41581440 | 10.29 | 32.13 |
| Medical induction of coma | 4 | 175775 | 302 | 41581230 | 3.13 | 5.73 |
| Drain placement | 4 | 175775 | 162 | 41581370 | 5.84 | 15.66 |
| Fluid intake restriction | 4 | 175775 | 66 | 41581466 | 14.34 | 46.79 |
| Blood test | 4 | 175775 | 124 | 41581408 | 7.63 | 22.33 |
| Arteriovenous fistula site haemorrhage | 4 | 175775 | 124 | 41581408 | 7.63 | 22.33 |
| Administration site erythema | 4 | 175775 | 221 | 41581311 | 4.28 | 9.88 |
| Anticoagulant therapy | 4 | 175775 | 94 | 41581438 | 10.07 | 31.33 |
| Vascular resistance pulmonary increased | 4 | 175775 | 57 | 41581475 | 16.6 | 54.8 |
| Accident at home | 4 | 175775 | 337 | 41581195 | 2.81 | 4.6 |
| Cirrhosis alcoholic | 4 | 175775 | 273 | 41581259 | 3.47 | 6.92 |
| Mitral valve repair | 4 | 175775 | 186 | 41581346 | 5.09 | 12.86 |
| Left ventricular end-diastolic pressure increased | 4 | 175775 | 119 | 41581413 | 7.95 | 23.52 |
| Ammonia abnormal | 4 | 175775 | 284 | 41581248 | 3.33 | 6.44 |
| Cranial operation | 4 | 175775 | 43 | 41581489 | 22.01 | 73.38 |
| Pericardial excision | 4 | 175775 | 52 | 41581480 | 18.2 | 60.36 |
| Rest regimen | 4 | 175775 | 247 | 41581285 | 3.83 | 8.23 |
| Oesophageal dilatation | 4 | 175775 | 277 | 41581255 | 3.42 | 6.74 |
| Cardiac failure high output | 4 | 175775 | 119 | 41581413 | 7.95 | 23.52 |
| Adenotonsillectomy | 4 | 175775 | 184 | 41581348 | 5.14 | 13.06 |

| | | | | | | |
|---|---|---|---|---|---|---|
| Eustachian tube disorder | 4 | 175775 | 144 | 41581388 | 6.57 | 18.38 |
| Pulmonary capillary haemangiomatosis | 4 | 175775 | 39 | 41581493 | 24.26 | 80.91 |
| Clostridial sepsis | 4 | 175775 | 230 | 41581302 | 4.11 | 9.27 |
| Elective surgery | 4 | 175775 | 132 | 41581400 | 7.17 | 20.61 |
| Human chorionic gonadotropin positive | 4 | 175775 | 39 | 41581493 | 24.26 | 80.91 |
| Exposure to fungus | 4 | 175775 | 235 | 41581297 | 4.03 | 8.95 |
| Enterocolitis viral | 4 | 175775 | 156 | 41581376 | 6.07 | 16.5 |
| Pulmonary vascular resistance abnormality | 4 | 175775 | 50 | 41581482 | 18.92 | 62.88 |
| Oesophageal operation | 4 | 175775 | 141 | 41581391 | 6.71 | 18.9 |
| Extubation | 4 | 175775 | 42 | 41581490 | 22.53 | 75.14 |
| Gastrointestinal angiodysplasia | 4 | 175775 | 279 | 41581253 | 3.39 | 6.65 |
| Vascular device occlusion | 4 | 175775 | 200 | 41581332 | 4.73 | 11.54 |
| Pregnancy test false positive | 4 | 175775 | 251 | 41581281 | 3.77 | 8.01 |
| Thoracic cavity drainage | 3 | 175776 | 169 | 41581363 | 4.2 | 7.18 |
| Venous operation | 3 | 175776 | 123 | 41581409 | 5.77 | 11.55 |
| Pulmonary arterial pressure | 3 | 175776 | 32 | 41581500 | 22.18 | 55.47 |
| Respiratory fume inhalation disorder | 3 | 175776 | 198 | 41581334 | 3.58 | 5.51 |
| Varicose vein ruptured | 3 | 175776 | 213 | 41581319 | 3.33 | 4.83 |
| Pulmonary function test | 3 | 175776 | 37 | 41581495 | 19.18 | 47.82 |
| Anomalous pulmonary venous connection | 3 | 175776 | 172 | 41581360 | 4.13 | 6.98 |
| Blood carbon monoxide increased | 3 | 175776 | 41 | 41581491 | 17.31 | 42.96 |
| Bronchoscopy | 3 | 175776 | 153 | 41581379 | 4.64 | 8.4 |
| Puncture site discharge | 3 | 175776 | 16 | 41581516 | 44.35 | 107.06 |
| Cardiac stress test | 3 | 175776 | 78 | 41581454 | 9.1 | 20.82 |
| Fistula repair | 3 | 175776 | 131 | 41581401 | 5.42 | 10.56 |
| Cardiac rehabilitation therapy | 3 | 175776 | 52 | 41581480 | 13.65 | 33.24 |
| Steroid therapy | 3 | 175776 | 155 | 41581377 | 4.58 | 8.23 |
| Contraception | 3 | 175776 | 11 | 41581521 | 64.52 | 147.39 |
| Tracheal haemorrhage | 3 | 175776 | 223 | 41581309 | 3.18 | 4.43 |
| Victim of abuse | 3 | 175776 | 124 | 41581408 | 5.72 | 11.42 |
| Shunt malfunction | 3 | 175776 | 168 | 41581364 | 4.22 | 7.25 |
| Administration site discharge | 3 | 175776 | 39 | 41581493 | 18.2 | 45.27 |
| Infective exacerbation of bronchiectasis | 3 | 175776 | 193 | 41581339 | 3.68 | 5.76 |
| Alcoholic | 3 | 175776 | 129 | 41581403 | 5.5 | 10.8 |

| | | | | | | |
|---|---|---|---|---|---|---|
| Pulmonary resection | 3 | 175776 | 186 | 41581346 | 3.82 | 6.13 |
| Cardiac pacemaker removal | 3 | 175776 | 45 | 41581487 | 15.77 | 38.91 |
| Splenic artery aneurysm | 3 | 175776 | 131 | 41581401 | 5.42 | 10.56 |
| Balloon atrial septostomy | 3 | 175776 | 39 | 41581493 | 18.2 | 45.27 |
| Catheter site erosion | 3 | 175776 | 75 | 41581457 | 9.46 | 21.83 |
| Graft infection | 3 | 175776 | 204 | 41581328 | 3.48 | 5.22 |
| Alcoholic liver disease | 3 | 175776 | 194 | 41581338 | 3.66 | 5.71 |
| Wound haematoma | 3 | 175776 | 118 | 41581414 | 6.01 | 12.23 |
| Device allergy | 3 | 175776 | 194 | 41581338 | 3.66 | 5.71 |
| Mass excision | 3 | 175776 | 225 | 41581307 | 3.15 | 4.36 |
| Anticoagulation drug level abnormal | 3 | 175776 | 85 | 41581447 | 8.35 | 18.74 |
| Pulmonary vein stenosis | 3 | 175776 | 87 | 41581445 | 8.16 | 18.21 |
| Biliary cirrhosis | 3 | 175776 | 95 | 41581437 | 7.47 | 16.3 |
| Pulmonary artery aneurysm | 3 | 175776 | 102 | 41581430 | 6.96 | 14.87 |
| Strangulated hernia | 3 | 175776 | 189 | 41581343 | 3.75 | 5.97 |
| Tracheostomy malfunction | 3 | 175776 | 140 | 41581392 | 5.07 | 9.59 |
| Probiotic therapy | 3 | 175776 | 84 | 41581448 | 8.45 | 19.02 |
| Thromboembolectomy | 3 | 175776 | 63 | 41581469 | 11.26 | 26.78 |
| Cardiac pacemaker adjustment | 3 | 175776 | 20 | 41581512 | 35.48 | 87.42 |
| Scleroderma associated digital ulcer | 3 | 175776 | 22 | 41581510 | 32.26 | 79.96 |
| Chronic gastrointestinal bleeding | 3 | 175776 | 214 | 41581318 | 3.32 | 4.79 |
| Chest tube insertion | 3 | 175776 | 171 | 41581361 | 4.15 | 7.05 |
| Wound closure | 3 | 175776 | 67 | 41581465 | 10.59 | 24.94 |
| Application site induration | 3 | 175776 | 133 | 41581399 | 5.34 | 10.34 |
| Pulmonary arteriopathy | 3 | 175776 | 30 | 41581502 | 23.66 | 59.18 |
| Intracardiac mass | 3 | 175776 | 122 | 41581410 | 5.82 | 11.68 |
| Arterial stent insertion | 3 | 175776 | 150 | 41581382 | 4.73 | 8.65 |
| Ventricular assist device insertion | 3 | 175776 | 61 | 41581471 | 11.63 | 27.79 |
| Oesophageal oedema | 3 | 175776 | 155 | 41581377 | 4.58 | 8.23 |
| Oesophageal injury | 3 | 175776 | 223 | 41581309 | 3.18 | 4.43 |
| Pulmonary artery therapeutic procedure | 3 | 175776 | 33 | 41581499 | 21.51 | 53.77 |
| Ventricular enlargement | 3 | 175776 | 153 | 41581379 | 4.64 | 8.4 |
| Arteriovenous fistula thrombosis | 3 | 175776 | 219 | 41581313 | 3.24 | 4.58 |
| Aspiration joint | 3 | 175776 | 124 | 41581408 | 5.72 | 11.42 |

| | | | | | | |
|---|---|---|---|---|---|---|
| Oxygen saturation | 3 | 175776 | 189 | 41581343 | 3.75 | 5.97 |
| Gastritis viral | 3 | 175776 | 87 | 41581445 | 8.16 | 18.21 |
| Procedural failure | 3 | 175776 | 112 | 41581420 | 6.34 | 13.13 |
| Blood bicarbonate increased | 3 | 175776 | 231 | 41581301 | 3.07 | 4.14 |
| Human metapneumovirus test positive | 3 | 175776 | 88 | 41581444 | 8.06 | 17.95 |
| Pregnancy test | 3 | 175776 | 7 | 41581525 | 101.38 | 208.72 |
| Arterial catheterisation | 3 | 175776 | 41 | 41581491 | 17.31 | 42.96 |
| Surgical procedure repeated | 3 | 175776 | 159 | 41581373 | 4.46 | 7.91 |
| Sensation of blood flow | 3 | 175776 | 228 | 41581304 | 3.11 | 4.25 |

Table S2： The proportion reporting ratio of the ADEs related to Bosentan.

| PT | a | b | c | d | PRR | χ2 |
|---|---|---|---|---|---|---|
| Death | 2142 | 56143 | 600376 | 41098650 | 2.55 | 2044.94 |
| Dyspnoea | 2097 | 56188 | 377960 | 41321066 | 3.97 | 4674.91 |
| Product dose omission issue | 1231 | 57054 | 374305 | 41324721 | 2.35 | 963.12 |
| Pneumonia | 930 | 57355 | 231108 | 41467918 | 2.88 | 1142.25 |
| Hospitalisation | 761 | 57524 | 109489 | 41589537 | 4.97 | 2404.86 |
| Cough | 613 | 57672 | 194764 | 41504262 | 2.25 | 427.22 |
| Pulmonary arterial hypertension | 606 | 57679 | 10949 | 41688077 | 39.6 | 21609.63 |
| Condition aggravated | 570 | 57715 | 203730 | 41495296 | 2 | 286.31 |
| Chest pain | 478 | 57807 | 112108 | 41586918 | 3.05 | 657.78 |
| Fluid retention | 463 | 57822 | 36609 | 41662417 | 9.05 | 3276 |
| Pulmonary hypertension | 395 | 57890 | 12962 | 41686064 | 21.8 | 7610.46 |
| Dyspnoea exertional | 390 | 57895 | 25318 | 41673708 | 11.02 | 3501.66 |
| Hypotension | 388 | 57897 | 131178 | 41567848 | 2.12 | 228.45 |
| Cardiac failure | 351 | 57934 | 51980 | 41647046 | 4.83 | 1060.52 |
| Syncope | 341 | 57944 | 63404 | 41635622 | 3.85 | 715.95 |
| Oxygen saturation decreased | 324 | 57961 | 36692 | 41662334 | 6.32 | 1438.73 |
| Oedema peripheral | 309 | 57976 | 61071 | 41637955 | 3.62 | 583.81 |
| Cardiac failure congestive | 296 | 57989 | 50144 | 41648882 | 4.22 | 724.76 |
| Atrial fibrillation | 288 | 57997 | 63386 | 41635640 | 3.25 | 447.43 |
| Palpitations | 287 | 57998 | 74895 | 41624131 | 2.74 | 316.87 |
| Disease progression | 278 | 58007 | 78517 | 41620509 | 2.53 | 257.52 |
| Influenza | 276 | 58009 | 77837 | 41621189 | 2.54 | 256.54 |

| | | | | | | |
|---|---|---|---|---|---|---|
| Hypoxia | 271 | 58014 | 21515 | 41677511 | 9.01 | 1907.17 |
| Respiratory failure | 252 | 58033 | 44498 | 41654528 | 4.05 | 576.56 |
| Right ventricular failure | 251 | 58034 | 4683 | 41694343 | 38.35 | 8665.96 |
| Adverse event | 244 | 58041 | 63641 | 41635385 | 2.74 | 269.62 |
| Alanine aminotransferase increased | 226 | 58059 | 32671 | 41666355 | 4.95 | 707.8 |
| Oedema | 223 | 58062 | 33815 | 41665211 | 4.72 | 649.64 |
| Pulmonary oedema | 218 | 58067 | 28181 | 41670845 | 5.53 | 804.22 |
| Nasal congestion | 217 | 58068 | 40516 | 41658510 | 3.83 | 452.15 |
| Aspartate aminotransferase increased | 216 | 58069 | 26899 | 41672127 | 5.74 | 840.31 |
| Cardiac disorder | 209 | 58076 | 60245 | 41638781 | 2.48 | 184.56 |
| Bronchitis | 190 | 58095 | 52692 | 41646334 | 2.58 | 183.38 |
| Liver function test increased | 183 | 58102 | 15769 | 41683257 | 8.3 | 1162.38 |
| Chronic obstructive pulmonary disease | 182 | 58103 | 34649 | 41664377 | 3.76 | 366.76 |
| Flushing | 171 | 58114 | 60175 | 41638851 | 2.03 | 89.64 |
| Transfusion | 170 | 58115 | 8327 | 41690699 | 14.61 | 2111.97 |
| Pain in jaw | 161 | 58124 | 19620 | 41679406 | 5.87 | 645.63 |
| Unevaluable event | 158 | 58127 | 55145 | 41643881 | 2.05 | 84.82 |
| Hypervolaemia | 155 | 58130 | 14684 | 41684342 | 7.55 | 872.18 |
| Respiratory distress | 143 | 58142 | 16534 | 41682492 | 6.19 | 616.86 |
| Cardiac arrest | 139 | 58146 | 48277 | 41650749 | 2.06 | 75.67 |
| Catheterisation cardiac | 137 | 58148 | 2938 | 41696088 | 33.36 | 4109.25 |
| Pleural effusion | 133 | 58152 | 38372 | 41660654 | 2.48 | 117.14 |
| Cardiac operation | 133 | 58152 | 5216 | 41693810 | 18.24 | 2113.91 |
| Lung disorder | 132 | 58153 | 31937 | 41667089 | 2.96 | 170.39 |
| Viral infection | 130 | 58155 | 21387 | 41677639 | 4.35 | 333.38 |
| Cellulitis | 122 | 58163 | 34527 | 41664499 | 2.53 | 112.37 |
| Haemoptysis | 115 | 58170 | 18551 | 41680475 | 4.44 | 304.21 |
| Acute respiratory failure | 113 | 58172 | 12629 | 41686397 | 6.4 | 510.6 |
| Lung transplant | 109 | 58176 | 2756 | 41696270 | 28.3 | 2761.05 |
| Productive cough | 107 | 58178 | 32883 | 41666143 | 2.33 | 80.86 |
| Blood alkaline phosphatase increased | 107 | 58178 | 12328 | 41686698 | 6.21 | 463.77 |
| Pericardial effusion | 104 | 58181 | 14389 | 41684637 | 5.17 | 347.5 |
| Cyanosis | 98 | 58187 | 8456 | 41690570 | 8.29 | 621.31 |
| Concomitant disease aggravated | 98 | 58187 | 4878 | 41694148 | 14.37 | 1195.52 |

| | | | | | | |
|---|---|---|---|---|---|---|
| Hepatic function abnormal | 94 | 58191 | 22072 | 41676954 | 3.05 | 128.78 |
| Device related infection | 93 | 58192 | 11642 | 41687384 | 5.72 | 359.01 |
| Upper respiratory tract infection | 90 | 58195 | 31801 | 41667225 | 2.02 | 46.58 |
| Ascites | 88 | 58197 | 18800 | 41680226 | 3.35 | 144.37 |
| Hospice care | 87 | 58198 | 7416 | 41691610 | 8.39 | 560.09 |
| Liver function test abnormal | 86 | 58199 | 15402 | 41683624 | 3.99 | 192.08 |
| Hip fracture | 83 | 58202 | 21219 | 41677807 | 2.8 | 95.61 |
| Seasonal allergy | 83 | 58202 | 10122 | 41688904 | 5.87 | 332.43 |
| Dialysis | 77 | 58208 | 7930 | 41691096 | 6.95 | 388.29 |
| Presyncope | 76 | 58209 | 16259 | 41682767 | 3.34 | 124.35 |
| Respiratory disorder | 76 | 58209 | 19236 | 41679790 | 2.83 | 89.4 |
| Gamma-glutamyltransferase increased | 75 | 58210 | 11637 | 41687389 | 4.61 | 210.79 |
| Skin ulcer | 75 | 58210 | 17549 | 41681477 | 3.06 | 103.45 |
| Rhinovirus infection | 74 | 58211 | 2438 | 41696588 | 21.72 | 1419.35 |
| Scleroderma | 73 | 58212 | 2439 | 41696587 | 21.41 | 1379.37 |
| Blood potassium decreased | 70 | 58215 | 18989 | 41680037 | 2.64 | 70.93 |
| Cardiac pacemaker insertion | 67 | 58218 | 3622 | 41695404 | 13.23 | 744.05 |
| Pulmonary arterial pressure increased | 67 | 58218 | 1121 | 41697905 | 42.76 | 2578.46 |
| Respiratory tract infection | 66 | 58219 | 17606 | 41681420 | 2.68 | 69.39 |
| Blood bilirubin increased | 66 | 58219 | 15094 | 41683932 | 3.13 | 95.18 |
| Localised infection | 66 | 58219 | 17827 | 41681199 | 2.65 | 67.51 |
| Infusion site pain | 63 | 58222 | 9177 | 41689849 | 4.91 | 194.95 |
| Staphylococcal infection | 62 | 58223 | 20103 | 41678923 | 2.21 | 40.79 |
| Head injury | 62 | 58223 | 20680 | 41678346 | 2.14 | 37.8 |
| Catheter site erythema | 62 | 58223 | 1473 | 41697553 | 30.11 | 1674.66 |
| Pneumothorax | 61 | 58224 | 10639 | 41688387 | 4.1 | 142.31 |
| Sinus disorder | 61 | 58224 | 14219 | 41684807 | 3.07 | 84.76 |
| Respiratory syncytial virus infection | 59 | 58226 | 5078 | 41693948 | 8.31 | 375.22 |
| Oxygen consumption increased | 58 | 58227 | 1782 | 41697244 | 23.29 | 1198.12 |
| Atrial flutter | 57 | 58228 | 4984 | 41694042 | 8.18 | 355.33 |
| Pulmonary congestion | 57 | 58228 | 7677 | 41691349 | 5.31 | 198.08 |
| Knee arthroplasty | 55 | 58230 | 13524 | 41685502 | 2.91 | 68.67 |
| Upper limb fracture | 55 | 58230 | 14364 | 41684662 | 2.74 | 60.53 |
| Catheter site pain | 55 | 58230 | 1713 | 41697313 | 22.97 | 1119.88 |

| | | | | | | |
|---|---|---|---|---|---|---|
| Catheter management | 55 | 58230 | 809 | 41698217 | 48.64 | 2402.96 |
| Heart transplant | 52 | 58233 | 535 | 41698491 | 69.54 | 3201.56 |
| Gout | 52 | 58233 | 12224 | 41686802 | 3.04 | 71.06 |
| Cardiomegaly | 52 | 58233 | 6334 | 41692692 | 5.87 | 208.59 |
| Disease complication | 50 | 58235 | 3335 | 41695691 | 10.73 | 434.49 |
| Catheter site infection | 48 | 58237 | 2638 | 41696388 | 13.02 | 523.06 |
| International normalised ratio increased | 47 | 58238 | 14661 | 41684365 | 2.29 | 34.19 |
| Gastroenteritis viral | 47 | 58238 | 12359 | 41686667 | 2.72 | 50.97 |
| Complication associated with device | 46 | 58239 | 15424 | 41683602 | 2.13 | 27.64 |
| Pneumonia aspiration | 46 | 58239 | 16077 | 41682949 | 2.05 | 24.57 |
| Concomitant disease progression | 46 | 58239 | 985 | 41698041 | 33.41 | 1381.79 |
| Rib fracture | 46 | 58239 | 14149 | 41684877 | 2.33 | 34.67 |
| Heart rate irregular | 45 | 58240 | 14317 | 41684709 | 2.25 | 31.12 |
| Hip arthroplasty | 43 | 58242 | 9862 | 41689164 | 3.12 | 61.67 |
| Lower limb fracture | 42 | 58243 | 12393 | 41686633 | 2.42 | 35.05 |
| Pulmonary hypertensive crisis | 41 | 58244 | 138 | 41698888 | 212.56 | 6655.64 |
| Pulmonary fibrosis | 41 | 58244 | 11446 | 41687580 | 2.56 | 38.94 |
| Haematocrit decreased | 41 | 58244 | 10827 | 41688199 | 2.71 | 44.06 |
| Internal haemorrhage | 41 | 58244 | 9892 | 41689134 | 2.97 | 53.2 |
| Stent placement | 41 | 58244 | 4942 | 41694084 | 5.94 | 166.89 |
| Therapy change | 40 | 58245 | 6703 | 41692323 | 4.27 | 99.56 |
| Exercise tolerance decreased | 39 | 58246 | 3930 | 41695096 | 7.1 | 202.39 |
| Respiratory tract congestion | 39 | 58246 | 10773 | 41688253 | 2.59 | 37.94 |
| Infusion site erythema | 39 | 58246 | 5580 | 41693446 | 5 | 123.96 |
| Product administered to patient of inappropriate age | 39 | 58246 | 10980 | 41688046 | 2.54 | 36.33 |
| Cardioversion | 38 | 58247 | 928 | 41698098 | 29.3 | 997.71 |
| Hepatic cirrhosis | 37 | 58248 | 11657 | 41687369 | 2.27 | 26.24 |
| Ankle fracture | 37 | 58248 | 10576 | 41688450 | 2.5 | 33.28 |
| Brain natriuretic peptide increased | 36 | 58249 | 1849 | 41697177 | 13.93 | 423.81 |
| Post procedural complication | 36 | 58249 | 10282 | 41688744 | 2.5 | 32.44 |
| Spinal operation | 36 | 58249 | 8318 | 41690708 | 3.1 | 50.89 |
| Tricuspid valve incompetence | 36 | 58249 | 3632 | 41695394 | 7.09 | 186.53 |
| Sudden death | 35 | 58250 | 5450 | 41693576 | 4.59 | 97.81 |
| Terminal state | 35 | 58250 | 5059 | 41693967 | 4.95 | 109.56 |

| | | | | | | |
|---|---|---|---|---|---|---|
| Secretion discharge | 34 | 58251 | 8863 | 41690163 | 2.74 | 37.57 |
| Catheter placement | 34 | 58251 | 1316 | 41697710 | 18.48 | 548.15 |
| Oxygen therapy | 34 | 58251 | 1596 | 41697430 | 15.24 | 443.01 |
| Gangrene | 34 | 58251 | 4040 | 41694986 | 6.02 | 141.19 |
| Sinus congestion | 34 | 58251 | 9084 | 41689942 | 2.68 | 35.62 |
| Tracheostomy | 33 | 58252 | 863 | 41698163 | 27.36 | 807.15 |
| Viral upper respiratory tract infection | 32 | 58253 | 4026 | 41695000 | 5.69 | 122.63 |
| Pulmonary haemorrhage | 31 | 58254 | 4822 | 41694204 | 4.6 | 86.77 |
| Bacteraemia | 31 | 58254 | 7510 | 41691516 | 2.95 | 39.89 |
| Cardiac ablation | 31 | 58254 | 1219 | 41697807 | 18.19 | 491.24 |
| Respiration abnormal | 31 | 58254 | 4807 | 41694219 | 4.61 | 87.19 |
| Cardiogenic shock | 30 | 58255 | 8610 | 41690416 | 2.49 | 26.73 |
| Right ventricular dysfunction | 30 | 58255 | 708 | 41698318 | 30.31 | 815.88 |
| Device occlusion | 30 | 58255 | 9341 | 41689685 | 2.3 | 21.92 |
| Hip surgery | 30 | 58255 | 3445 | 41695581 | 6.23 | 130.59 |
| Hepatomegaly | 29 | 58256 | 5606 | 41693420 | 3.7 | 56.88 |
| Catheter site haemorrhage | 29 | 58256 | 1601 | 41697425 | 12.96 | 314.37 |
| Supraventricular tachycardia | 28 | 58257 | 5563 | 41693463 | 3.6 | 52.35 |
| Knee operation | 28 | 58257 | 6296 | 41692730 | 3.18 | 41.71 |
| Tooth extraction | 28 | 58257 | 7250 | 41691776 | 2.76 | 31.38 |
| Oxygen saturation abnormal | 28 | 58257 | 1917 | 41697109 | 10.45 | 235.84 |
| Dyspnoea at rest | 28 | 58257 | 2113 | 41696913 | 9.48 | 209.64 |
| Cardiac failure acute | 28 | 58257 | 4335 | 41694691 | 4.62 | 78.95 |
| Generalised oedema | 27 | 58258 | 6983 | 41692043 | 2.77 | 30.34 |
| Systemic scleroderma | 27 | 58258 | 554 | 41698472 | 34.87 | 846.94 |
| Blood iron decreased | 27 | 58258 | 8211 | 41690815 | 2.35 | 20.93 |
| Hernia repair | 27 | 58258 | 1667 | 41697359 | 11.59 | 257.05 |
| Gastrointestinal tube insertion | 27 | 58258 | 1463 | 41697563 | 13.2 | 299.03 |
| Skin infection | 26 | 58259 | 7870 | 41691156 | 2.36 | 20.39 |
| Catheter site swelling | 26 | 58259 | 644 | 41698382 | 28.88 | 672.73 |
| Carbon dioxide increased | 26 | 58259 | 873 | 41698153 | 21.31 | 488.67 |
| Infusion site swelling | 25 | 58260 | 4273 | 41694753 | 4.19 | 60.27 |
| Cataract operation | 25 | 58260 | 3727 | 41695299 | 4.8 | 74.69 |
| Bedridden | 25 | 58260 | 8721 | 41690305 | 2.05 | 13.43 |

| | | | | | | |
|---|---|---|---|---|---|---|
| Transplant evaluation | 25 | 58260 | 425 | 41698601 | 42.08 | 947.01 |
| Haemodynamic instability | 25 | 58260 | 4445 | 41694581 | 4.02 | 56.5 |
| Right-to-left cardiac shunt | 24 | 58261 | 162 | 41698864 | 105.99 | 2173.94 |
| Living in residential institution | 24 | 58261 | 1364 | 41697662 | 12.59 | 251.61 |
| Orthopnoea | 23 | 58262 | 1933 | 41697093 | 8.51 | 150.71 |
| Leg amputation | 23 | 58262 | 2599 | 41696427 | 6.33 | 102.35 |
| Finger amputation | 23 | 58262 | 435 | 41698591 | 37.83 | 783.24 |
| Dizziness postural | 23 | 58262 | 6200 | 41692826 | 2.65 | 23.62 |
| Vascular device infection | 23 | 58262 | 3248 | 41695778 | 5.07 | 74.54 |
| Aspiration | 23 | 58262 | 6222 | 41692804 | 2.64 | 23.44 |
| Left ventricular failure | 23 | 58262 | 1991 | 41697035 | 8.26 | 145.2 |
| Pulmonary thrombosis | 23 | 58262 | 7634 | 41691392 | 2.16 | 14.21 |
| Cholecystectomy | 23 | 58262 | 6409 | 41692617 | 2.57 | 21.93 |
| Respiratory tract infection viral | 22 | 58263 | 1488 | 41697538 | 10.58 | 188.02 |
| Angioplasty | 22 | 58263 | 791 | 41698235 | 19.9 | 384.19 |
| Catheter site pruritus | 22 | 58263 | 736 | 41698290 | 21.39 | 415.1 |
| Pelvic fracture | 22 | 58263 | 5517 | 41693509 | 2.85 | 26.37 |
| Rehabilitation therapy | 22 | 58263 | 2821 | 41696205 | 5.58 | 82.06 |
| Catheter site discharge | 21 | 58264 | 759 | 41698267 | 19.79 | 364.66 |
| Raynaud's phenomenon | 21 | 58264 | 3166 | 41695860 | 4.75 | 61.68 |
| Aortic stenosis | 21 | 58264 | 2058 | 41696968 | 7.3 | 113.04 |
| Pneumonia bacterial | 21 | 58264 | 5860 | 41693166 | 2.56 | 19.96 |
| Iron deficiency anaemia | 20 | 58265 | 5999 | 41693027 | 2.39 | 16.04 |
| Cardiac flutter | 20 | 58265 | 4158 | 41694868 | 3.44 | 34.47 |
| Poor peripheral circulation | 20 | 58265 | 2836 | 41696190 | 5.05 | 64.42 |
| Back injury | 20 | 58265 | 6809 | 41692217 | 2.1 | 11.51 |
| Red blood cell transfusion | 20 | 58265 | 1395 | 41697631 | 10.26 | 164.74 |
| Transplant | 20 | 58265 | 1630 | 41697396 | 8.78 | 136.18 |
| Mechanical ventilation | 19 | 58266 | 1093 | 41697933 | 12.44 | 196.41 |
| Endotracheal intubation | 19 | 58266 | 1739 | 41697287 | 7.82 | 111.73 |
| Iron deficiency | 19 | 58266 | 2999 | 41696027 | 4.53 | 51.99 |
| Ulcer haemorrhage | 19 | 58266 | 4514 | 41694512 | 3.01 | 25.42 |
| Urine output decreased | 19 | 58266 | 5174 | 41693852 | 2.63 | 19.08 |
| Tracheitis | 19 | 58266 | 476 | 41698550 | 28.56 | 485.86 |

| | | | | | | |
|---|---|---|---|---|---|---|
| Toe amputation | 19 | 58266 | 3968 | 41695058 | 3.43 | 32.48 |
| Atrial septal defect repair | 19 | 58266 | 113 | 41698913 | 120.29 | 1924.21 |
| Pulmonary veno-occlusive disease | 19 | 58266 | 452 | 41698574 | 30.07 | 512.49 |
| Chronic respiratory failure | 19 | 58266 | 751 | 41698275 | 18.1 | 299.38 |
| Pulmonary vein stenosis | 19 | 58266 | 71 | 41698955 | 191.45 | 2839.79 |
| Limb operation | 18 | 58267 | 3215 | 41695811 | 4.01 | 40.37 |
| Cardiac murmur | 18 | 58267 | 4502 | 41694524 | 2.86 | 21.7 |
| Multiple allergies | 18 | 58267 | 5490 | 41693536 | 2.35 | 13.85 |
| Atelectasis | 18 | 58267 | 5324 | 41693702 | 2.42 | 14.93 |
| Gastrostomy | 18 | 58267 | 1076 | 41697950 | 11.97 | 177.96 |
| Localised oedema | 17 | 58268 | 3042 | 41695984 | 4 | 38.01 |
| Mastectomy | 17 | 58268 | 1001 | 41698025 | 12.15 | 171.05 |
| Hysterectomy | 17 | 58268 | 5400 | 41693626 | 2.25 | 11.8 |
| Bronchiolitis | 17 | 58268 | 2182 | 41696844 | 5.57 | 63.32 |
| Pulseless electrical activity | 17 | 58268 | 3012 | 41696014 | 4.04 | 38.64 |
| Muscle strain | 17 | 58268 | 5988 | 41693038 | 2.03 | 8.87 |
| Sarcoidosis | 17 | 58268 | 2975 | 41696051 | 4.09 | 39.44 |
| N-terminal prohormone brain natriuretic peptide increased | 17 | 58268 | 973 | 41698053 | 12.5 | 176.77 |
| Dependence on oxygen therapy | 17 | 58268 | 315 | 41698711 | 38.61 | 590.94 |
| Infusion site infection | 16 | 58269 | 1525 | 41697501 | 7.51 | 89.3 |
| Heart disease congenital | 16 | 58269 | 2389 | 41696637 | 4.79 | 47.69 |
| Pneumonia viral | 16 | 58269 | 2167 | 41696859 | 5.28 | 55.14 |
| Bilirubin conjugated increased | 16 | 58269 | 1186 | 41697840 | 9.65 | 122.44 |
| Biopsy liver | 16 | 58269 | 268 | 41698758 | 42.71 | 615.06 |
| Walking distance test abnormal | 16 | 58269 | 297 | 41698729 | 38.54 | 555.18 |
| Eye operation | 16 | 58269 | 2839 | 41696187 | 4.03 | 36.28 |
| Pulmonary arterial pressure abnormal | 16 | 58269 | 204 | 41698822 | 56.11 | 803.1 |
| Haemodialysis | 16 | 58269 | 3193 | 41695833 | 3.59 | 29.68 |
| Failure to thrive | 16 | 58269 | 2947 | 41696079 | 3.88 | 34.08 |
| Metapneumovirus infection | 15 | 58270 | 486 | 41698540 | 22.08 | 292.86 |
| Implantable defibrillator insertion | 15 | 58270 | 754 | 41698272 | 14.23 | 180.95 |
| Catheter site inflammation | 15 | 58270 | 419 | 41698607 | 25.61 | 342.51 |
| Rales | 15 | 58270 | 3671 | 41695355 | 2.92 | 18.91 |
| Pseudomonas infection | 15 | 58270 | 4989 | 41694037 | 2.15 | 9.21 |

| | | | | | | |
|---|---|---|---|---|---|---|
| Autoimmune hepatitis | 15 | 58270 | 4348 | 41694678 | 2.47 | 13.06 |
| Haemolytic anaemia | 15 | 58270 | 5214 | 41693812 | 2.06 | 8.14 |
| Troponin increased | 15 | 58270 | 4359 | 41694667 | 2.46 | 12.98 |
| Pulmonary alveolar haemorrhage | 15 | 58270 | 3420 | 41695606 | 3.14 | 21.75 |
| Product supply issue | 15 | 58270 | 5034 | 41693992 | 2.13 | 8.99 |
| Coronary arterial stent insertion | 14 | 58271 | 3569 | 41695457 | 2.81 | 16.22 |
| Right ventricular systolic pressure increased | 14 | 58271 | 170 | 41698856 | 58.92 | 736.45 |
| Cardiac failure chronic | 14 | 58271 | 3157 | 41695869 | 3.17 | 20.74 |
| Staphylococcal sepsis | 14 | 58271 | 2776 | 41696250 | 3.61 | 26.26 |
| Heart rate abnormal | 14 | 58271 | 3813 | 41695213 | 2.63 | 14.05 |
| Heart and lung transplant | 14 | 58271 | 84 | 41698942 | 119.24 | 1406.97 |
| Hypercapnia | 14 | 58271 | 1811 | 41697215 | 5.53 | 51.56 |
| Vein disorder | 14 | 58271 | 3457 | 41695569 | 2.9 | 17.33 |
| Choking sensation | 14 | 58271 | 3546 | 41695480 | 2.82 | 16.44 |
| Patent ductus arteriosus repair | 14 | 58271 | 19 | 41699007 | 527.16 | 4233.14 |
| Enterovirus infection | 14 | 58271 | 594 | 41698432 | 16.86 | 204.09 |
| Procedural complication | 13 | 58272 | 3773 | 41695253 | 2.47 | 11.28 |
| Heart valve incompetence | 13 | 58272 | 1946 | 41697080 | 4.78 | 38.6 |
| Right ventricular hypertrophy | 13 | 58272 | 596 | 41698430 | 15.61 | 173.91 |
| Echocardiogram abnormal | 13 | 58272 | 689 | 41698337 | 13.5 | 147.66 |
| Bronchopulmonary dysplasia | 13 | 58272 | 407 | 41698619 | 22.85 | 263.24 |
| Aspiration pleural cavity | 13 | 58272 | 566 | 41698460 | 16.43 | 184.18 |
| Pulmonary pain | 13 | 58272 | 2246 | 41696780 | 4.14 | 30.8 |
| Right ventricular dilatation | 13 | 58272 | 456 | 41698570 | 20.4 | 233.14 |
| Emergency care | 13 | 58272 | 2013 | 41697013 | 4.62 | 36.64 |
| Heart valve replacement | 12 | 58273 | 953 | 41698073 | 9.01 | 84.37 |
| Food poisoning | 12 | 58273 | 3504 | 41695522 | 2.45 | 10.26 |
| Cor pulmonale | 12 | 58273 | 536 | 41698490 | 16.02 | 165.26 |
| Chemotherapy | 12 | 58273 | 2328 | 41696698 | 3.69 | 23.39 |
| Staphylococcus test positive | 12 | 58273 | 1503 | 41697523 | 5.71 | 46.28 |
| Shoulder operation | 12 | 58273 | 2726 | 41696300 | 3.15 | 17.53 |
| Infusion site discharge | 12 | 58273 | 879 | 41698147 | 9.77 | 93.16 |
| Pleurisy | 12 | 58273 | 3642 | 41695384 | 2.36 | 9.35 |
| Brain abscess | 12 | 58273 | 1583 | 41697443 | 5.42 | 42.97 |

| | | | | | | |
|---|---|---|---|---|---|---|
| Peripheral ischaemia | 12 | 58273 | 2432 | 41696594 | 3.53 | 21.65 |
| Balloon atrial septostomy | 12 | 58273 | 30 | 41698996 | 286.17 | 2435.8 |
| Parainfluenzae virus infection | 12 | 58273 | 1119 | 41697907 | 7.67 | 68.89 |
| Device related sepsis | 11 | 58274 | 1541 | 41697485 | 5.11 | 36.07 |
| Cardiopulmonary failure | 11 | 58274 | 2292 | 41696734 | 3.43 | 18.88 |
| Cardiac pacemaker replacement | 11 | 58274 | 338 | 41698688 | 23.28 | 227.2 |
| Renal transplant | 11 | 58274 | 2161 | 41696865 | 3.64 | 20.97 |
| Tracheostomy infection | 11 | 58274 | 76 | 41698950 | 103.55 | 975.9 |
| Blood culture positive | 11 | 58274 | 1038 | 41697988 | 7.58 | 62.19 |
| Infusion site haemorrhage | 11 | 58274 | 2322 | 41696704 | 3.39 | 18.44 |
| Aortic valve replacement | 11 | 58274 | 709 | 41698317 | 11.1 | 99.55 |
| Paracentesis | 11 | 58274 | 459 | 41698567 | 17.15 | 163.33 |
| Endocarditis | 11 | 58274 | 3203 | 41695823 | 2.46 | 9.47 |
| Device alarm issue | 11 | 58274 | 1436 | 41697590 | 5.48 | 39.99 |
| Humerus fracture | 11 | 58274 | 3207 | 41695819 | 2.45 | 9.44 |
| Angiopathy | 11 | 58274 | 3167 | 41695859 | 2.48 | 9.73 |
| Breath sounds abnormal | 11 | 58274 | 3308 | 41695718 | 2.38 | 8.76 |
| Polycythaemia | 11 | 58274 | 1116 | 41697910 | 7.05 | 56.57 |
| Blood pressure systolic decreased | 11 | 58274 | 2745 | 41696281 | 2.87 | 13.32 |
| Administration site pain | 11 | 58274 | 758 | 41698268 | 10.38 | 91.93 |
| Normal newborn | 10 | 58275 | 3188 | 41695838 | 2.24 | 6.88 |
| Pulse absent | 10 | 58275 | 2372 | 41696654 | 3.02 | 13.42 |
| Paranasal sinus hypersecretion | 10 | 58275 | 2673 | 41696353 | 2.68 | 10.46 |
| Catheter site rash | 10 | 58275 | 362 | 41698664 | 19.76 | 173.35 |
| Varicose vein | 10 | 58275 | 3549 | 41695477 | 2.02 | 5.11 |
| Paranasal sinus discomfort | 10 | 58275 | 2870 | 41696156 | 2.49 | 8.91 |
| Device infusion issue | 10 | 58275 | 1750 | 41697276 | 4.09 | 23.2 |
| Tonsillectomy | 10 | 58275 | 1188 | 41697838 | 6.02 | 41.53 |
| Aneurysm | 10 | 58275 | 3090 | 41695936 | 2.32 | 7.45 |
| Ocular icterus | 10 | 58275 | 2537 | 41696489 | 2.82 | 11.7 |
| Clavicle fracture | 10 | 58275 | 2648 | 41696378 | 2.7 | 10.68 |
| Atrial tachycardia | 10 | 58275 | 1311 | 41697715 | 5.46 | 36.13 |
| Ischaemia | 10 | 58275 | 2792 | 41696234 | 2.56 | 9.49 |
| Therapeutic embolisation | 10 | 58275 | 216 | 41698810 | 33.12 | 297.74 |

| | | | | | | |
|---|---|---|---|---|---|---|
| Human metapneumovirus test positive | 10 | 58275 | 81 | 41698945 | 88.33 | 768.49 |
| Infusion site irritation | 9 | 58276 | 820 | 41698206 | 7.85 | 53.23 |
| Congestive hepatopathy | 9 | 58276 | 696 | 41698330 | 9.25 | 65.39 |
| Craniofacial fracture | 9 | 58276 | 2951 | 41696075 | 2.18 | 5.75 |
| Necrosis | 9 | 58276 | 3193 | 41695833 | 2.02 | 4.6 |
| Lung assist device therapy | 9 | 58276 | 61 | 41698965 | 105.56 | 812.25 |
| Catheter site related reaction | 9 | 58276 | 381 | 41698645 | 16.9 | 131.53 |
| Central venous catheterisation | 9 | 58276 | 2307 | 41696719 | 2.79 | 10.3 |
| Mitral valve repair | 9 | 58276 | 181 | 41698845 | 35.57 | 288.1 |
| International normalised ratio abnormal | 9 | 58276 | 2071 | 41696955 | 3.11 | 12.82 |
| Pulmonary artery therapeutic procedure | 9 | 58276 | 27 | 41698999 | 238.48 | 1596.25 |
| Extremity necrosis | 9 | 58276 | 1138 | 41697888 | 5.66 | 34.24 |
| Biopsy lung | 9 | 58276 | 266 | 41698760 | 24.21 | 193.68 |
| Rectal prolapse | 9 | 58276 | 792 | 41698234 | 8.13 | 55.65 |
| Compression fracture | 9 | 58276 | 2786 | 41696240 | 2.31 | 6.67 |
| Tracheostomy malfunction | 9 | 58276 | 134 | 41698892 | 48.05 | 388.56 |
| Primary biliary cholangitis | 9 | 58276 | 464 | 41698562 | 13.88 | 105.5 |
| Ventricular assist device insertion | 9 | 58276 | 55 | 41698971 | 117.07 | 890.07 |
| Peripheral arterial occlusive disease | 9 | 58276 | 2814 | 41696212 | 2.29 | 6.51 |
| Walking disability | 9 | 58276 | 1524 | 41697502 | 4.22 | 22.03 |
| Cardiac output decreased | 9 | 58276 | 508 | 41698518 | 12.67 | 95.1 |
| Ventricular dysfunction | 9 | 58276 | 874 | 41698152 | 7.37 | 49.02 |
| Biopsy | 9 | 58276 | 578 | 41698448 | 11.14 | 81.8 |
| Azotaemia | 8 | 58277 | 2131 | 41696895 | 2.69 | 8.43 |
| Colon operation | 8 | 58277 | 717 | 41698309 | 7.98 | 48.32 |
| Functional gastrointestinal disorder | 8 | 58277 | 2782 | 41696244 | 2.06 | 4.33 |
| Mineral supplementation | 8 | 58277 | 363 | 41698663 | 15.77 | 108.26 |
| Oesophageal haemorrhage | 8 | 58277 | 843 | 41698183 | 6.79 | 39.12 |
| Tracheal disorder | 8 | 58277 | 316 | 41698710 | 18.11 | 126.15 |
| Scleroderma associated digital ulcer | 8 | 58277 | 17 | 41699009 | 336.67 | 1820.65 |
| Scleroderma renal crisis | 8 | 58277 | 246 | 41698780 | 23.27 | 165.1 |
| Respiratory acidosis | 8 | 58277 | 2403 | 41696623 | 2.38 | 6.39 |
| Sickle cell anaemia | 8 | 58277 | 437 | 41698589 | 13.1 | 87.78 |
| Plasmapheresis | 8 | 58277 | 342 | 41698684 | 16.74 | 115.66 |

| | | | | | | |
|---|---|---|---|---|---|---|
| Infusion site pruritus | 8 | 58277 | 2129 | 41696897 | 2.69 | 8.45 |
| Arteriovenous fistula operation | 8 | 58277 | 223 | 41698803 | 25.67 | 183.07 |
| Face injury | 8 | 58277 | 2365 | 41696661 | 2.42 | 6.64 |
| Resuscitation | 8 | 58277 | 802 | 41698224 | 7.14 | 41.8 |
| Clubbing | 8 | 58277 | 252 | 41698774 | 22.71 | 160.94 |
| Catheter site warmth | 8 | 58277 | 95 | 41698931 | 60.25 | 429.91 |
| Infusion site rash | 8 | 58277 | 926 | 41698100 | 6.18 | 34.44 |
| Peripheral artery occlusion | 8 | 58277 | 1260 | 41697766 | 4.54 | 21.96 |
| Device connection issue | 8 | 58277 | 1123 | 41697903 | 5.1 | 26.16 |
| Pulmonary artery dilatation | 8 | 58277 | 204 | 41698822 | 28.06 | 200.86 |
| Use of accessory respiratory muscles | 8 | 58277 | 239 | 41698787 | 23.95 | 170.22 |
| Diagnostic procedure | 8 | 58277 | 115 | 41698911 | 49.77 | 357.45 |
| Feeding intolerance | 8 | 58277 | 348 | 41698678 | 16.45 | 113.45 |
| Heart sounds abnormal | 8 | 58277 | 438 | 41698588 | 13.07 | 87.55 |
| Liver transplant | 8 | 58277 | 2035 | 41696991 | 2.81 | 9.31 |
| Low cardiac output syndrome | 8 | 58277 | 222 | 41698804 | 25.78 | 183.93 |
| Pericardial drainage | 8 | 58277 | 153 | 41698873 | 37.41 | 269.4 |
| Exposure to sars-cov-2 | 8 | 58277 | 1596 | 41697430 | 3.59 | 14.85 |
| Radiotherapy | 7 | 58278 | 1179 | 41697847 | 4.25 | 17.28 |
| Medical device change | 7 | 58278 | 349 | 41698677 | 14.35 | 85.23 |
| Oesophageal candidiasis | 7 | 58278 | 2347 | 41696679 | 2.13 | 4.2 |
| Cavopulmonary anastomosis | 7 | 58278 | 3 | 41699023 | 1669.34 | 3501.42 |
| Craniocerebral injury | 7 | 58278 | 2263 | 41696763 | 2.21 | 4.64 |
| Colostomy | 7 | 58278 | 1256 | 41697770 | 3.99 | 15.58 |
| Nocturnal dyspnoea | 7 | 58278 | 833 | 41698193 | 6.01 | 29.01 |
| Biopsy bone marrow | 7 | 58278 | 345 | 41698681 | 14.52 | 86.34 |
| Systemic-pulmonary artery shunt | 7 | 58278 | 20 | 41699006 | 250.4 | 1288.03 |
| Injection site infection | 7 | 58278 | 2084 | 41696942 | 2.4 | 5.72 |
| Aortic valve incompetence | 7 | 58278 | 2025 | 41697001 | 2.47 | 6.12 |
| Catheter site vesicles | 7 | 58278 | 146 | 41698880 | 34.3 | 215.96 |
| Infusion site warmth | 7 | 58278 | 840 | 41698186 | 5.96 | 28.67 |
| Pulmonary arterial wedge pressure increased | 7 | 58278 | 91 | 41698935 | 55.03 | 344.84 |
| Diastolic dysfunction | 7 | 58278 | 1805 | 41697221 | 2.77 | 7.91 |
| Mitral valve replacement | 7 | 58278 | 211 | 41698815 | 23.73 | 147.54 |

| | | | | | | |
|---|---|---|---|---|---|---|
| Non-cardiac chest pain | 7 | 58278 | 1805 | 41697221 | 2.77 | 7.91 |
| Ear congestion | 7 | 58278 | 1850 | 41697176 | 2.71 | 7.51 |
| Polypectomy | 7 | 58278 | 502 | 41698524 | 9.98 | 55.76 |
| Sinus operation | 7 | 58278 | 1629 | 41697397 | 3.07 | 9.76 |
| Amputation | 7 | 58278 | 828 | 41698198 | 6.05 | 29.25 |
| Medical device implantation | 7 | 58278 | 889 | 41698137 | 5.63 | 26.47 |
| Medical procedure | 7 | 58278 | 951 | 41698075 | 5.27 | 24.02 |
| Colonoscopy | 7 | 58278 | 1477 | 41697549 | 3.39 | 11.74 |
| Chest injury | 7 | 58278 | 1488 | 41697538 | 3.37 | 11.59 |
| Lyme disease | 7 | 58278 | 2335 | 41696691 | 2.14 | 4.26 |
| Rhonchi | 7 | 58278 | 801 | 41698225 | 6.25 | 30.62 |
| Catheter removal | 7 | 58278 | 271 | 41698755 | 18.48 | 112.82 |
| Ventricular hypertrophy | 7 | 58278 | 626 | 41698400 | 8 | 42.4 |
| Foot amputation | 7 | 58278 | 1063 | 41697963 | 4.71 | 20.33 |
| Pulmonary artery banding | 7 | 58278 | 5 | 41699021 | 1001.61 | 2915.52 |
| Heart valve operation | 7 | 58278 | 290 | 41698736 | 17.27 | 104.76 |
| Patella fracture | 7 | 58278 | 1561 | 41697465 | 3.21 | 10.59 |
| Tachyarrhythmia | 7 | 58278 | 1483 | 41697543 | 3.38 | 11.66 |
| Trisomy 21 | 7 | 58278 | 739 | 41698287 | 6.78 | 34.15 |
| Protein-losing gastroenteropathy | 7 | 58278 | 301 | 41698725 | 16.64 | 100.55 |
| Lung opacity | 7 | 58278 | 1616 | 41697410 | 3.1 | 9.91 |
| Intellectual disability | 7 | 58278 | 1352 | 41697674 | 3.7 | 13.75 |
| Large intestinal haemorrhage | 6 | 58279 | 1503 | 41697523 | 2.86 | 7.21 |
| Cancer surgery | 6 | 58279 | 783 | 41698243 | 5.48 | 21.82 |
| Middle ear effusion | 6 | 58279 | 1515 | 41697511 | 2.83 | 7.09 |
| Brain operation | 6 | 58279 | 1504 | 41697522 | 2.85 | 7.2 |
| Vascular operation | 6 | 58279 | 255 | 41698771 | 16.83 | 87.31 |
| Cardiovascular insufficiency | 6 | 58279 | 1083 | 41697943 | 3.96 | 13.22 |
| Cor pulmonale chronic | 6 | 58279 | 121 | 41698905 | 35.48 | 191.53 |
| Sudden cardiac death | 6 | 58279 | 1700 | 41697326 | 2.53 | 5.51 |
| Occult blood positive | 6 | 58279 | 1150 | 41697876 | 3.73 | 11.94 |
| Adenotonsillectomy | 6 | 58279 | 182 | 41698844 | 23.59 | 125.63 |
| Crest syndrome | 6 | 58279 | 169 | 41698857 | 25.4 | 135.81 |
| Platelet transfusion | 6 | 58279 | 958 | 41698068 | 4.48 | 16.12 |

| | | | | | | |
|---|---|---|---|---|---|---|
| Hypertrophic cardiomyopathy | 6 | 58279 | 915 | 41698111 | 4.69 | 17.31 |
| Blood potassium abnormal | 6 | 58279 | 1803 | 41697223 | 2.38 | 4.79 |
| Ventricular septal defect repair | 6 | 58279 | 20 | 41699006 | 214.63 | 981.39 |
| Vocal cord paralysis | 6 | 58279 | 1115 | 41697911 | 3.85 | 12.59 |
| Portal hypertension | 6 | 58279 | 1823 | 41697203 | 2.35 | 4.66 |
| Cyst removal | 6 | 58279 | 360 | 41698666 | 11.92 | 59.06 |
| Myocardial necrosis marker increased | 6 | 58279 | 1272 | 41697754 | 3.37 | 9.98 |
| Organ failure | 6 | 58279 | 1879 | 41697147 | 2.28 | 4.32 |
| Pulmonary resection | 6 | 58279 | 183 | 41698843 | 23.46 | 124.9 |
| Dental care | 6 | 58279 | 1423 | 41697603 | 3.02 | 8.05 |
| Neck surgery | 6 | 58279 | 1665 | 41697361 | 2.58 | 5.78 |
| Vascular graft | 6 | 58279 | 1851 | 41697175 | 2.32 | 4.49 |
| Biliary colic | 6 | 58279 | 1856 | 41697170 | 2.31 | 4.46 |
| Rotator cuff repair | 6 | 58279 | 1152 | 41697874 | 3.73 | 11.91 |
| Therapeutic procedure | 6 | 58279 | 839 | 41698187 | 5.12 | 19.73 |
| Gastrostomy tube site complication | 6 | 58279 | 146 | 41698880 | 29.4 | 158.11 |
| Infected skin ulcer | 6 | 58279 | 1322 | 41697704 | 3.25 | 9.29 |
| Platelet disorder | 6 | 58279 | 1775 | 41697251 | 2.42 | 4.97 |
| Positive airway pressure therapy | 6 | 58279 | 403 | 41698623 | 10.65 | 51.7 |
| Brain death | 6 | 58279 | 1739 | 41697287 | 2.47 | 5.22 |
| Pulmonary artery stenosis | 6 | 58279 | 289 | 41698737 | 14.85 | 75.95 |
| Blood chloride decreased | 6 | 58279 | 1060 | 41697966 | 4.05 | 13.7 |
| Catheter site irritation | 6 | 58279 | 226 | 41698800 | 18.99 | 99.63 |
| Right ventricular enlargement | 6 | 58279 | 231 | 41698795 | 18.58 | 97.29 |
| Cardiac resynchronisation therapy | 6 | 58279 | 50 | 41698976 | 85.85 | 449.27 |
| Thrombectomy | 6 | 58279 | 249 | 41698777 | 17.24 | 89.63 |
| Menopause | 6 | 58279 | 1579 | 41697447 | 2.72 | 6.49 |
| Chronic respiratory disease | 6 | 58279 | 228 | 41698798 | 18.83 | 98.69 |
| Biopsy kidney | 5 | 58280 | 179 | 41698847 | 19.98 | 87.72 |
| Atrioventricular septal defect | 5 | 58280 | 338 | 41698688 | 10.58 | 42.76 |
| Pupil fixed | 5 | 58280 | 747 | 41698279 | 4.79 | 14.89 |
| Lymphadenectomy | 5 | 58280 | 629 | 41698397 | 5.69 | 19.16 |
| Pulmonary function test abnormal | 5 | 58280 | 1188 | 41697838 | 3.01 | 6.69 |
| Traumatic lung injury | 5 | 58280 | 1341 | 41697685 | 2.67 | 5.19 |

| | | | | | | |
|---|---|---|---|---|---|---|
| Tumour excision | 5 | 58280 | 531 | 41698495 | 6.74 | 24.2 |
| Fracture treatment | 5 | 58280 | 120 | 41698906 | 29.81 | 133.65 |
| Procalcitonin increased | 5 | 58280 | 813 | 41698213 | 4.4 | 13.06 |
| Abdominal cavity drainage | 5 | 58280 | 319 | 41698707 | 11.21 | 45.8 |
| Total lung capacity decreased | 5 | 58280 | 1093 | 41697933 | 3.27 | 7.86 |
| Oesophageal rupture | 5 | 58280 | 517 | 41698509 | 6.92 | 25.08 |
| Pulmonary vascular disorder | 5 | 58280 | 302 | 41698724 | 11.84 | 48.84 |
| Right atrial dilatation | 5 | 58280 | 286 | 41698740 | 12.51 | 52.03 |
| Gastric operation | 5 | 58280 | 560 | 41698466 | 6.39 | 22.52 |
| Laparotomy | 5 | 58280 | 299 | 41698727 | 11.96 | 49.41 |
| Increased bronchial secretion | 5 | 58280 | 1392 | 41697634 | 2.57 | 4.78 |
| Abdominal operation | 5 | 58280 | 750 | 41698276 | 4.77 | 14.8 |
| Aspartate aminotransferase abnormal | 5 | 58280 | 863 | 41698163 | 4.15 | 11.86 |
| Haematoma evacuation | 5 | 58280 | 191 | 41698835 | 18.73 | 81.77 |
| Teething | 5 | 58280 | 203 | 41698823 | 17.62 | 76.51 |
| Ankle operation | 5 | 58280 | 949 | 41698077 | 3.77 | 10.12 |
| Catheter site abscess | 5 | 58280 | 107 | 41698919 | 33.43 | 150.28 |
| Aortic valve disease | 5 | 58280 | 974 | 41698052 | 3.67 | 9.68 |
| Diuretic therapy | 5 | 58280 | 55 | 41698971 | 65.04 | 289 |
| Dermatitis diaper | 5 | 58280 | 458 | 41698568 | 7.81 | 29.37 |
| Cardiac septal defect repair | 5 | 58280 | 21 | 41699005 | 170.34 | 679.86 |
| Microcytic anaemia | 5 | 58280 | 1308 | 41697718 | 2.73 | 5.48 |
| Dysentery | 5 | 58280 | 1418 | 41697608 | 2.52 | 4.58 |
| Cholelithotomy | 5 | 58280 | 139 | 41698887 | 25.74 | 114.74 |
| Infusion site reaction | 5 | 58280 | 1463 | 41697563 | 2.45 | 4.26 |
| Bladder spasm | 5 | 58280 | 1110 | 41697916 | 3.22 | 7.63 |
| Breast operation | 5 | 58280 | 424 | 41698602 | 8.44 | 32.39 |
| Oesophageal motility disorder | 5 | 58280 | 261 | 41698765 | 13.71 | 57.79 |
| Pulmonary hypoplasia | 5 | 58280 | 476 | 41698550 | 7.52 | 27.95 |
| Serratia infection | 5 | 58280 | 372 | 41698654 | 9.62 | 38.09 |
| Vasodilation procedure | 5 | 58280 | 23 | 41699003 | 155.53 | 630.59 |
| Pulmonary endarterectomy | 5 | 58280 | 128 | 41698898 | 27.95 | 125.03 |
| Skin neoplasm excision | 5 | 58280 | 516 | 41698510 | 6.93 | 25.14 |
| Pancreatic mass | 5 | 58280 | 708 | 41698318 | 5.05 | 16.14 |

| | | | | | | |
|---|---|---|---|---|---|---|
| Diet noncompliance | 5 | 58280 | 290 | 41698736 | 12.34 | 51.2 |
| Arterial stent insertion | 5 | 58280 | 148 | 41698878 | 24.17 | 107.43 |
| Pneumonia influenzal | 5 | 58280 | 756 | 41698270 | 4.73 | 14.62 |
| Palliative care | 5 | 58280 | 1015 | 41698011 | 3.52 | 9 |
| Coccydynia | 5 | 58280 | 1079 | 41697947 | 3.32 | 8.05 |
| Pneumonia staphylococcal | 5 | 58280 | 1053 | 41697973 | 3.4 | 8.42 |
| Oesophagogastric fundoplasty | 5 | 58280 | 91 | 41698935 | 39.31 | 176.95 |
| Pulmonary artery aneurysm | 5 | 58280 | 100 | 41698926 | 35.77 | 160.95 |
| Irregular breathing | 5 | 58280 | 461 | 41698565 | 7.76 | 29.13 |
| Oral surgery | 5 | 58280 | 1492 | 41697534 | 2.4 | 4.06 |
| Dyspnoea paroxysmal nocturnal | 5 | 58280 | 394 | 41698632 | 9.08 | 35.5 |
| Dengue fever | 5 | 58280 | 1476 | 41697550 | 2.42 | 4.17 |
| Infusion | 5 | 58280 | 314 | 41698712 | 11.39 | 46.66 |
| Critical illness | 5 | 58280 | 517 | 41698509 | 6.92 | 25.08 |
| Endarterectomy | 5 | 58280 | 160 | 41698866 | 22.36 | 98.92 |
| Pulmonary valve incompetence | 5 | 58280 | 648 | 41698378 | 5.52 | 18.37 |
| Capillary leak syndrome | 5 | 58280 | 1210 | 41697816 | 2.96 | 6.45 |
| Drainage | 5 | 58280 | 292 | 41698734 | 12.25 | 50.79 |
| Coccidioidomycosis | 4 | 58281 | 788 | 41698238 | 3.63 | 7.59 |
| Mitral valve disease | 4 | 58281 | 1006 | 41698020 | 2.84 | 4.77 |
| Tricuspid valve repair | 4 | 58281 | 23 | 41699003 | 124.42 | 417.17 |
| Dialysis related complication | 4 | 58281 | 285 | 41698741 | 10.04 | 32.11 |
| Suture rupture | 4 | 58281 | 483 | 41698543 | 5.92 | 16.24 |
| Infusion site abscess | 4 | 58281 | 218 | 41698808 | 13.13 | 44.01 |
| Intestinal polypectomy | 4 | 58281 | 28 | 41698998 | 102.2 | 350.75 |
| Intestinal mass | 4 | 58281 | 956 | 41698070 | 2.99 | 5.29 |
| Traumatic haematoma | 4 | 58281 | 746 | 41698280 | 3.84 | 8.34 |
| Suture insertion | 4 | 58281 | 342 | 41698684 | 8.37 | 25.65 |
| Exercise test abnormal | 4 | 58281 | 25 | 41699001 | 114.47 | 387.86 |
| Aneurysm repair | 4 | 58281 | 80 | 41698946 | 35.77 | 128.76 |
| Groin infection | 4 | 58281 | 393 | 41698633 | 7.28 | 21.46 |
| Infusion site vesicles | 4 | 58281 | 392 | 41698634 | 7.3 | 21.53 |
| Acute right ventricular failure | 4 | 58281 | 110 | 41698916 | 26.02 | 92.84 |
| Coagulation time prolonged | 4 | 58281 | 897 | 41698129 | 3.19 | 5.99 |

| | | | | | | |
|---|---|---|---|---|---|---|
| Prostatic operation | 4 | 58281 | 697 | 41698329 | 4.11 | 9.34 |
| Blood electrolytes decreased | 4 | 58281 | 905 | 41698121 | 3.16 | 5.89 |
| Jejunostomy | 4 | 58281 | 121 | 41698905 | 23.65 | 84 |
| Application site hypersensitivity | 4 | 58281 | 850 | 41698176 | 3.37 | 6.62 |
| Bronchial obstruction | 4 | 58281 | 907 | 41698119 | 3.16 | 5.86 |
| Bone operation | 4 | 58281 | 369 | 41698657 | 7.76 | 23.29 |
| Gastrointestinal bacterial infection | 4 | 58281 | 830 | 41698196 | 3.45 | 6.92 |
| Stem cell transplant | 4 | 58281 | 1029 | 41697997 | 2.78 | 4.55 |
| Haemodynamic test abnormal | 4 | 58281 | 45 | 41698981 | 63.59 | 226.32 |
| Oesophageal dilation procedure | 4 | 58281 | 102 | 41698924 | 28.06 | 100.43 |
| Pulmonary sepsis | 4 | 58281 | 1039 | 41697987 | 2.75 | 4.45 |
| Congenital diaphragmatic hernia | 4 | 58281 | 251 | 41698775 | 11.4 | 37.36 |
| Breast conserving surgery | 4 | 58281 | 538 | 41698488 | 5.32 | 13.93 |
| Serositis | 4 | 58281 | 277 | 41698749 | 10.33 | 33.23 |
| Phlebotomy | 4 | 58281 | 54 | 41698972 | 53 | 189.98 |
| Pseudomonal sepsis | 4 | 58281 | 1023 | 41698003 | 2.8 | 4.6 |
| Bladder operation | 4 | 58281 | 770 | 41698256 | 3.72 | 7.9 |
| Catheterisation cardiac abnormal | 4 | 58281 | 132 | 41698894 | 21.68 | 76.58 |
| Computerised tomogram thorax abnormal | 4 | 58281 | 571 | 41698455 | 5.01 | 12.76 |
| Adenoidectomy | 4 | 58281 | 247 | 41698779 | 11.59 | 38.07 |
| Renal stone removal | 4 | 58281 | 361 | 41698665 | 7.93 | 23.95 |
| Vascular resistance pulmonary increased | 4 | 58281 | 57 | 41698969 | 50.21 | 180.25 |
| Croup infectious | 4 | 58281 | 505 | 41698521 | 5.67 | 15.25 |
| Poor feeding infant | 4 | 58281 | 853 | 41698173 | 3.35 | 6.58 |
| Transfusion reaction | 4 | 58281 | 456 | 41698570 | 6.28 | 17.59 |
| Liver function test | 4 | 58281 | 58 | 41698968 | 49.34 | 177.22 |
| Respiratory syncytial virus bronchiolitis | 4 | 58281 | 374 | 41698652 | 7.65 | 22.88 |
| Exposure to fungus | 4 | 58281 | 235 | 41698791 | 12.18 | 40.35 |
| Implantable defibrillator replacement | 4 | 58281 | 37 | 41698989 | 77.34 | 272.02 |
| Blood culture negative | 4 | 58281 | 57 | 41698969 | 50.21 | 180.25 |
| Micrococcus test positive | 4 | 58281 | 18 | 41699008 | 158.99 | 513.79 |
| Fractured coccyx | 4 | 58281 | 851 | 41698175 | 3.36 | 6.61 |
| Mitral valve calcification | 4 | 58281 | 283 | 41698743 | 10.11 | 32.39 |
| Alanine aminotransferase | 4 | 58281 | 131 | 41698895 | 21.85 | 77.21 |

| | | | | | | |
|---|---|---|---|---|---|---|
| Acute left ventricular failure | 4 | 58281 | 477 | 41698549 | 6 | 16.53 |
| Bronchitis viral | 4 | 58281 | 468 | 41698558 | 6.11 | 16.97 |
| Prinzmetal angina | 4 | 58281 | 594 | 41698432 | 4.82 | 12.02 |
| Aortic valve repair | 4 | 58281 | 54 | 41698972 | 53 | 189.98 |
| Collateral circulation | 4 | 58281 | 93 | 41698933 | 30.77 | 110.46 |
| Fontan procedure | 4 | 58281 | 4 | 41699022 | 715.43 | 1426.87 |
| Pulmonary vein occlusion | 4 | 58281 | 61 | 41698965 | 46.91 | 168.68 |
| Lung transplant rejection | 4 | 58281 | 394 | 41698632 | 7.26 | 21.39 |
| Arterial aneurysm repair | 4 | 58281 | 6 | 41699020 | 476.96 | 1139.9 |
| Spinal cord operation | 4 | 58281 | 115 | 41698911 | 24.88 | 88.62 |
| Graft haemorrhage | 4 | 58281 | 48 | 41698978 | 59.62 | 212.81 |
| Dependence on respirator | 4 | 58281 | 268 | 41698758 | 10.68 | 34.57 |
| Therapeutic product effective for unapproved indication | 4 | 58281 | 269 | 41698757 | 10.64 | 34.42 |
| Hilar lymphadenopathy | 4 | 58281 | 512 | 41698514 | 5.59 | 14.96 |
| Mitral valve stenosis | 3 | 58282 | 356 | 41698670 | 6.03 | 12.48 |
| Shunt malfunction | 3 | 58282 | 168 | 41698858 | 12.78 | 31.99 |
| Colon neoplasm | 3 | 58282 | 647 | 41698379 | 3.32 | 4.83 |
| Craniotomy | 3 | 58282 | 275 | 41698751 | 7.8 | 17.61 |
| Hypoplastic left heart syndrome | 3 | 58282 | 402 | 41698624 | 5.34 | 10.5 |
| Tracheal haemorrhage | 3 | 58282 | 223 | 41698803 | 9.62 | 22.88 |
| Catheter site discolouration | 3 | 58282 | 77 | 41698949 | 27.87 | 74.81 |
| Infusion site mass | 3 | 58282 | 708 | 41698318 | 3.03 | 4.07 |
| Pulmonary sarcoidosis | 3 | 58282 | 638 | 41698388 | 3.36 | 4.96 |
| Hydrothorax | 3 | 58282 | 600 | 41698426 | 3.58 | 5.54 |
| Chest tube insertion | 3 | 58282 | 171 | 41698855 | 12.55 | 31.34 |
| Gastrointestinal surgery | 3 | 58282 | 511 | 41698515 | 4.2 | 7.27 |
| Jugular vein distension | 3 | 58282 | 329 | 41698697 | 6.52 | 13.9 |
| Osteotomy | 3 | 58282 | 137 | 41698889 | 15.67 | 40.31 |
| Blood alkaline phosphatase abnormal | 3 | 58282 | 548 | 41698478 | 3.92 | 6.48 |
| Cardiopulmonary bypass | 3 | 58282 | 15 | 41699011 | 143.09 | 352.73 |
| Pneumonia escherichia | 3 | 58282 | 212 | 41698814 | 10.12 | 24.32 |
| Cardiac procedure complication | 3 | 58282 | 314 | 41698712 | 6.84 | 14.8 |
| Abscess drainage | 3 | 58282 | 459 | 41698567 | 4.68 | 8.61 |
| Thyrotoxic crisis | 3 | 58282 | 679 | 41698347 | 3.16 | 4.41 |

| | | | | | | |
|---|---|---|---|---|---|---|
| Tracheostomy tube removal | 3 | 58282 | 19 | 41699007 | 112.96 | 287.52 |
| Wrist surgery | 3 | 58282 | 645 | 41698381 | 3.33 | 4.86 |
| Pulmonary valve replacement | 3 | 58282 | 16 | 41699010 | 134.14 | 333.86 |
| Vascular cauterisation | 3 | 58282 | 50 | 41698976 | 42.93 | 115.89 |
| Joint surgery | 3 | 58282 | 270 | 41698756 | 7.95 | 18.03 |
| Fear of falling | 3 | 58282 | 585 | 41698441 | 3.67 | 5.79 |
| Inferior vena cava dilatation | 3 | 58282 | 153 | 41698873 | 14.03 | 35.6 |
| Catheter site induration | 3 | 58282 | 45 | 41698981 | 47.7 | 128.58 |
| Enterovirus test positive | 3 | 58282 | 121 | 41698905 | 17.74 | 46.24 |
| Venous operation | 3 | 58282 | 123 | 41698903 | 17.45 | 45.41 |
| Blood gases abnormal | 3 | 58282 | 278 | 41698748 | 7.72 | 17.36 |
| Endotracheal intubation complication | 3 | 58282 | 346 | 41698680 | 6.2 | 12.98 |
| Inguinal hernia repair | 3 | 58282 | 227 | 41698799 | 9.46 | 22.39 |
| Infusion site inflammation | 3 | 58282 | 363 | 41698663 | 5.91 | 12.15 |
| Incorrect product dosage form administered | 3 | 58282 | 658 | 41698368 | 3.26 | 4.68 |
| Hepatorenal failure | 3 | 58282 | 321 | 41698705 | 6.69 | 14.37 |
| Lower respiratory tract infection viral | 3 | 58282 | 371 | 41698655 | 5.79 | 11.78 |
| Gastrointestinal bacterial overgrowth | 3 | 58282 | 666 | 41698360 | 3.22 | 4.58 |
| Laryngeal stenosis | 3 | 58282 | 370 | 41698656 | 5.8 | 11.82 |
| Medical device site infection | 3 | 58282 | 507 | 41698519 | 4.23 | 7.37 |
| Pneumonia respiratory syncytial viral | 3 | 58282 | 517 | 41698509 | 4.15 | 7.14 |
| Cardiac failure high output | 3 | 58282 | 120 | 41698906 | 17.89 | 46.66 |
| Scrotal swelling | 3 | 58282 | 599 | 41698427 | 3.58 | 5.56 |
| Large intestinal polypectomy | 3 | 58282 | 154 | 41698872 | 13.94 | 35.34 |
| Cardiac infection | 3 | 58282 | 537 | 41698489 | 4 | 6.7 |
| Arteriovenous malformation | 3 | 58282 | 565 | 41698461 | 3.8 | 6.15 |
| Gastrointestinal polyp haemorrhage | 3 | 58282 | 499 | 41698527 | 4.3 | 7.56 |
| Aspartate aminotransferase | 3 | 58282 | 160 | 41698866 | 13.41 | 33.83 |
| Inner ear disorder | 3 | 58282 | 638 | 41698388 | 3.36 | 4.96 |
| Chronic gastrointestinal bleeding | 3 | 58282 | 214 | 41698812 | 10.03 | 24.05 |
| Right atrial pressure increased | 3 | 58282 | 49 | 41698977 | 43.8 | 118.24 |
| Infusion site cellulitis | 3 | 58282 | 308 | 41698718 | 6.97 | 15.19 |
| Systemic sclerosis pulmonary | 3 | 58282 | 37 | 41698989 | 58.01 | 155.47 |
| Thyroxine free increased | 3 | 58282 | 438 | 41698588 | 4.9 | 9.25 |

| | | | | | | |
|---|---|---|---|---|---|---|
| Spinal laminectomy | 3 | 58282 | 634 | 41698392 | 3.39 | 5.02 |
| Renal haematoma | 3 | 58282 | 373 | 41698653 | 5.75 | 11.69 |
| Sclerodactylia | 3 | 58282 | 58 | 41698968 | 37.01 | 99.93 |
| Tricuspid valve replacement | 3 | 58282 | 35 | 41698991 | 61.32 | 163.96 |
| Respiratory tract infection bacterial | 3 | 58282 | 384 | 41698642 | 5.59 | 11.22 |
| Excessive granulation tissue | 3 | 58282 | 531 | 41698495 | 4.04 | 6.83 |
| Adenoidal disorder | 3 | 58282 | 165 | 41698861 | 13.01 | 32.66 |
| Surgical procedure repeated | 3 | 58282 | 159 | 41698867 | 13.5 | 34.08 |
| Catheter site mass | 3 | 58282 | 57 | 41698969 | 37.65 | 101.69 |
| Orthopaedic procedure | 3 | 58282 | 380 | 41698646 | 5.65 | 11.39 |
| Human rhinovirus test positive | 3 | 58282 | 203 | 41698823 | 10.57 | 25.62 |
| Right atrial enlargement | 3 | 58282 | 158 | 41698868 | 13.58 | 34.32 |
| Cardiorenal syndrome | 3 | 58282 | 449 | 41698577 | 4.78 | 8.91 |
| Ventricular failure | 3 | 58282 | 184 | 41698842 | 11.66 | 28.78 |
| Transcatheter aortic valve implantation | 3 | 58282 | 85 | 41698941 | 25.25 | 67.49 |
| Sleep study | 3 | 58282 | 51 | 41698975 | 42.08 | 113.64 |
| Rotavirus infection | 3 | 58282 | 345 | 41698681 | 6.22 | 13.03 |
| Vocal cord operation | 3 | 58282 | 59 | 41698967 | 36.38 | 98.22 |
| Sickle cell disease | 3 | 58282 | 410 | 41698616 | 5.23 | 10.2 |
| Vascular stent insertion | 3 | 58282 | 71 | 41698955 | 30.23 | 81.35 |
| Haemophilus infection | 3 | 58282 | 562 | 41698464 | 3.82 | 6.21 |
| Pseudomonas test positive | 3 | 58282 | 549 | 41698477 | 3.91 | 6.46 |
| Skin operation | 3 | 58282 | 197 | 41698829 | 10.89 | 26.56 |
| Cyanosis central | 3 | 58282 | 161 | 41698865 | 13.33 | 33.59 |
| Cardiac valve vegetation | 3 | 58282 | 245 | 41698781 | 8.76 | 20.37 |
| Bladder catheterisation | 3 | 58282 | 599 | 41698427 | 3.58 | 5.56 |
| Cranial operation | 3 | 58282 | 44 | 41698982 | 48.78 | 131.44 |
| Sinus rhythm | 3 | 58282 | 317 | 41698709 | 6.77 | 14.62 |
| Cardiac index decreased | 3 | 58282 | 44 | 41698982 | 48.78 | 131.44 |
| Prostatectomy | 3 | 58282 | 322 | 41698704 | 6.67 | 14.31 |
| Vasospasm | 3 | 58282 | 486 | 41698540 | 4.42 | 7.88 |
| Respiratory tract haemorrhage | 3 | 58282 | 426 | 41698600 | 5.04 | 9.64 |
| Ventilation perfusion mismatch | 3 | 58282 | 194 | 41698832 | 11.06 | 27.04 |
| Lung hyperinflation | 3 | 58282 | 618 | 41698408 | 3.47 | 5.26 |

| | | | | | | |
|---|---|---|---|---|---|---|
| Streptococcal bacteraemia | 3 | 58282 | 669 | 41698357 | 3.21 | 4.54 |
| Oxygen consumption | 3 | 58282 | 129 | 41698897 | 16.64 | 43.09 |
| Vein collapse | 3 | 58282 | 633 | 41698393 | 3.39 | 5.03 |
| Electrocardiogram qrs complex abnormal | 3 | 58282 | 190 | 41698836 | 11.3 | 27.72 |
| Pericardiotomy | 3 | 58282 | 3 | 41699023 | 715.43 | 1070.15 |
| Prohormone brain natriuretic peptide increased | 3 | 58282 | 62 | 41698964 | 34.62 | 93.42 |
| Peripheral circulatory failure | 3 | 58282 | 271 | 41698755 | 7.92 | 17.94 |
| Congenital arterial malformation | 3 | 58282 | 126 | 41698900 | 17.03 | 44.23 |
| Shoulder fracture | 3 | 58282 | 472 | 41698554 | 4.55 | 8.25 |

Table S3: The proportion reporting ratio of the ADEs related to Iloprost.

| PT | a | b | c | d | PRR | $\chi2$ |
|---|---|---|---|---|---|---|
| Death | 1194 | 11878 | 601324 | 41142915 | 6.34 | 5439.16 |
| Dyspnoea | 455 | 12617 | 379602 | 41364637 | 3.83 | 958.05 |
| Hospitalisation | 208 | 12864 | 110042 | 41634197 | 6.04 | 874.64 |
| Cough | 194 | 12878 | 195183 | 41549056 | 3.17 | 289.96 |
| Pulmonary arterial hypertension | 368 | 25776 | 24544 | 83463934 | 47.88 | 16647.08 |
| Pneumonia | 151 | 12921 | 231887 | 41512352 | 2.08 | 85.03 |
| Product use issue | 140 | 12932 | 150565 | 41593674 | 2.97 | 183.35 |
| Inappropriate schedule of product administration | 139 | 12933 | 184355 | 41559884 | 2.41 | 114.83 |
| Fluid retention | 129 | 12943 | 36943 | 41707296 | 11.15 | 1188.95 |
| Chest pain | 122 | 12950 | 112464 | 41631775 | 3.46 | 214.19 |
| Oedema peripheral | 118 | 12954 | 61262 | 41682977 | 6.15 | 508.77 |
| Hypotension | 111 | 12961 | 131455 | 41612784 | 2.7 | 118.75 |
| Syncope | 103 | 12969 | 63642 | 41680597 | 5.17 | 346.23 |
| Cardiac failure | 102 | 12970 | 52229 | 41692010 | 6.24 | 448.17 |
| Oxygen saturation decreased | 95 | 12977 | 36921 | 41707318 | 8.22 | 601.15 |
| General physical health deterioration | 88 | 12984 | 71558 | 41672681 | 3.93 | 192.09 |
| Disease progression | 80 | 12992 | 78715 | 41665524 | 3.25 | 124.4 |
| Cardiac arrest | 73 | 12999 | 48343 | 41695896 | 4.82 | 221.08 |
| Oedema | 67 | 13005 | 33971 | 41710268 | 6.3 | 298.28 |
| Respiratory failure | 64 | 13008 | 44686 | 41699553 | 4.57 | 178.64 |
| Influenza | 63 | 13009 | 78050 | 41666189 | 2.58 | 60.9 |
| Loss of consciousness | 54 | 13018 | 76639 | 41667600 | 2.25 | 37.55 |

| | | | | | | |
|---|---|---|---|---|---|---|
| Epistaxis | 54 | 13018 | 52515 | 41691724 | 3.28 | 85.79 |
| Cardiac failure congestive | 54 | 13018 | 50386 | 41693853 | 3.42 | 92.6 |
| Right ventricular failure | 52 | 13020 | 4882 | 41739357 | 34.01 | 1648.9 |
| Dyspnoea exertional | 51 | 13021 | 25657 | 41718582 | 6.35 | 229.45 |
| Cardio-respiratory arrest | 49 | 13023 | 24296 | 41719943 | 6.44 | 224.87 |
| Lung disorder | 46 | 13026 | 32023 | 41712216 | 4.59 | 128.95 |
| Pulmonary oedema | 43 | 13029 | 28356 | 41715883 | 4.84 | 131 |
| Tachycardia | 43 | 13029 | 55061 | 41689178 | 2.49 | 38.5 |
| Cardiac disorder | 42 | 13030 | 60412 | 41683827 | 2.22 | 28.18 |
| Bronchitis | 41 | 13031 | 52841 | 41691398 | 2.48 | 36.15 |
| Productive cough | 38 | 13034 | 32952 | 41711287 | 3.68 | 74.23 |
| Chronic obstructive pulmonary disease | 34 | 13038 | 34797 | 41709442 | 3.12 | 48.98 |
| Hypoxia | 31 | 13041 | 21755 | 41722484 | 4.55 | 85.8 |
| Throat irritation | 31 | 13041 | 29665 | 41714574 | 3.34 | 50.72 |
| Pleural effusion | 31 | 13041 | 38474 | 41705765 | 2.57 | 29.82 |
| Transfusion | 29 | 13043 | 8468 | 41735771 | 10.94 | 260.96 |
| Hypervolaemia | 29 | 13043 | 14810 | 41729429 | 6.25 | 127.77 |
| Therapy non-responder | 28 | 13044 | 37103 | 41707136 | 2.41 | 23.1 |
| Arrhythmia | 28 | 13044 | 28597 | 41715642 | 3.13 | 40.49 |
| Treatment noncompliance | 28 | 13044 | 33252 | 41710987 | 2.69 | 29.7 |
| Haemoptysis | 28 | 13044 | 18638 | 41725601 | 4.8 | 84.08 |
| Pain in jaw | 28 | 13044 | 19753 | 41724486 | 4.53 | 76.86 |
| Respiratory distress | 27 | 13045 | 16650 | 41727589 | 5.18 | 90.92 |
| Nasal congestion | 26 | 13046 | 40707 | 41703532 | 2.04 | 13.78 |
| Asphyxia | 26 | 13046 | 5687 | 41738552 | 14.6 | 327.92 |
| Drug dose omission by device | 25 | 13047 | 35750 | 41708489 | 2.23 | 17.03 |
| Choking | 24 | 13048 | 13014 | 41731225 | 5.89 | 97.27 |
| Respiratory arrest | 24 | 13048 | 14080 | 41730159 | 5.44 | 86.93 |
| Product prescribing issue | 24 | 13048 | 10146 | 41734093 | 7.55 | 136.18 |
| Sudden death | 22 | 13050 | 5463 | 41738776 | 12.86 | 239.7 |
| Renal disorder | 22 | 13050 | 31949 | 41712290 | 2.2 | 14.38 |
| Lung transplant | 21 | 13051 | 2844 | 41741395 | 23.58 | 450.77 |
| Labelled drug-drug interaction medication error | 21 | 13051 | 8174 | 41736065 | 8.2 | 132.53 |
| Pulmonary arterial pressure increased | 21 | 13051 | 1167 | 41743072 | 57.46 | 1144.57 |

| | | | | | | |
|---|---|---|---|---|---|---|
| Ascites | 21 | 13051 | 18867 | 41725372 | 3.55 | 38.53 |
| Device use error | 21 | 13051 | 26257 | 41717982 | 2.55 | 19.85 |
| Exercise tolerance decreased | 20 | 13052 | 3949 | 41740290 | 16.17 | 283.29 |
| Mouth haemorrhage | 20 | 13052 | 4667 | 41739572 | 13.69 | 234.19 |
| Respiratory disorder | 20 | 13052 | 19292 | 41724947 | 3.31 | 32.23 |
| Product administered to patient of inappropriate age | 20 | 13052 | 10999 | 41733240 | 5.81 | 79.46 |
| Heart rate decreased | 19 | 13053 | 25150 | 41719089 | 2.41 | 15.71 |
| Head injury | 19 | 13053 | 20723 | 41723516 | 2.93 | 24.11 |
| Acute respiratory failure | 19 | 13053 | 12723 | 41731516 | 4.77 | 56.53 |
| Premature baby | 18 | 13054 | 21439 | 41722800 | 2.68 | 18.97 |
| Caesarean section | 17 | 13055 | 5756 | 41738483 | 9.43 | 127.78 |
| Premature delivery | 17 | 13055 | 13278 | 41730961 | 4.09 | 39.63 |
| Drug delivery system malfunction | 17 | 13055 | 2842 | 41741397 | 19.1 | 289.91 |
| Intestinal obstruction | 16 | 13056 | 24403 | 41719836 | 2.09 | 9.14 |
| Pulmonary fibrosis | 16 | 13056 | 11471 | 41732768 | 4.45 | 42.81 |
| Cardiac pacemaker insertion | 16 | 13056 | 3673 | 41740566 | 13.91 | 190.91 |
| Choking sensation | 16 | 13056 | 3544 | 41740695 | 14.42 | 198.9 |
| Generalised oedema | 15 | 13057 | 6995 | 41737244 | 6.85 | 74.76 |
| Haematemesis | 15 | 13057 | 15811 | 41728428 | 3.03 | 20.38 |
| Gout | 14 | 13058 | 12262 | 41731977 | 3.65 | 26.86 |
| Hepatic cirrhosis | 13 | 13059 | 11681 | 41732558 | 3.55 | 23.84 |
| Presyncope | 13 | 13059 | 16322 | 41727917 | 2.54 | 12.17 |
| Respiratory tract infection | 13 | 13059 | 17659 | 41726580 | 2.35 | 10.09 |
| Circulatory collapse | 13 | 13059 | 10074 | 41734165 | 4.12 | 30.69 |
| Scleroderma | 13 | 13059 | 2499 | 41741740 | 16.61 | 189.77 |
| Low birth weight baby | 13 | 13059 | 6528 | 41737711 | 6.36 | 58.61 |
| Respiratory tract congestion | 13 | 13059 | 10799 | 41733440 | 3.84 | 27.33 |
| Disease complication | 13 | 13059 | 3372 | 41740867 | 12.31 | 134.6 |
| Pericardial effusion | 13 | 13059 | 14480 | 41729759 | 2.87 | 15.8 |
| Cardiomegaly | 13 | 13059 | 6373 | 41737866 | 6.51 | 60.56 |
| Catheterisation cardiac | 12 | 13060 | 3063 | 41741176 | 12.51 | 126.6 |
| Cholelithiasis | 12 | 13060 | 17768 | 41726471 | 2.16 | 7.44 |
| Localised infection | 12 | 13060 | 17881 | 41726358 | 2.14 | 7.31 |
| Incorrect dose administered by device | 12 | 13060 | 15802 | 41728437 | 2.43 | 10.05 |

| | | | | | | |
|---|---|---|---|---|---|---|
| Cyanosis | 11 | 13061 | 8543 | 41735696 | 4.11 | 25.88 |
| Sleep apnoea syndrome | 11 | 13061 | 13616 | 41730623 | 2.58 | 10.64 |
| Poor quality sleep | 11 | 13061 | 14745 | 41729494 | 2.38 | 8.82 |
| Concomitant disease aggravated | 11 | 13061 | 4965 | 41739274 | 7.08 | 57.26 |
| Dialysis | 10 | 13062 | 7997 | 41736242 | 3.99 | 22.41 |
| Oral pain | 10 | 13062 | 15704 | 41728535 | 2.03 | 5.25 |
| Dry throat | 10 | 13062 | 6890 | 41737349 | 4.63 | 28.47 |
| Drug administered in wrong device | 10 | 13062 | 627 | 41743612 | 50.93 | 481.83 |
| Pulmonary thrombosis | 9 | 13063 | 7648 | 41736591 | 3.76 | 18.2 |
| Kidney infection | 9 | 13063 | 14007 | 41730232 | 2.05 | 4.85 |
| Internal haemorrhage | 9 | 13063 | 9924 | 41734315 | 2.9 | 11.16 |
| Atrial flutter | 9 | 13063 | 5032 | 41739207 | 5.71 | 34.92 |
| Nasal discomfort | 9 | 13063 | 5293 | 41738946 | 5.43 | 32.48 |
| Head discomfort | 9 | 13063 | 13194 | 41731045 | 2.18 | 5.73 |
| Chemotherapy | 8 | 13064 | 2332 | 41741907 | 10.96 | 72.13 |
| Cardiopulmonary failure | 8 | 13064 | 2295 | 41741944 | 11.13 | 73.52 |
| Device failure | 8 | 13064 | 10701 | 41733538 | 2.39 | 6.45 |
| Investigation | 8 | 13064 | 2993 | 41741246 | 8.54 | 53.08 |
| Subdural haematoma | 8 | 13064 | 10038 | 41734201 | 2.55 | 7.5 |
| Cardiovascular disorder | 8 | 13064 | 12306 | 41731933 | 2.08 | 4.46 |
| Blood loss anaemia | 8 | 13064 | 5253 | 41738986 | 4.86 | 24.52 |
| Suffocation feeling | 8 | 13064 | 1560 | 41742679 | 16.38 | 114.92 |
| Hyperthyroidism | 8 | 13064 | 9441 | 41734798 | 2.71 | 8.6 |
| Cardiac operation | 8 | 13064 | 5341 | 41738898 | 4.78 | 23.91 |
| Device use issue | 8 | 13064 | 11876 | 41732363 | 2.15 | 4.93 |
| Ammonia increased | 7 | 13065 | 3235 | 41741004 | 6.91 | 35.31 |
| Secretion discharge | 7 | 13065 | 8890 | 41735349 | 2.51 | 6.38 |
| Intestinal haemorrhage | 7 | 13065 | 3789 | 41740450 | 5.9 | 28.43 |
| Blood potassium increased | 7 | 13065 | 9945 | 41734294 | 2.25 | 4.85 |
| Oral discomfort | 7 | 13065 | 9218 | 41735021 | 2.43 | 5.86 |
| Small intestinal obstruction | 7 | 13065 | 7809 | 41736430 | 2.86 | 8.48 |
| Viral upper respiratory tract infection | 7 | 13065 | 4051 | 41740188 | 5.52 | 25.85 |
| Rhinalgia | 7 | 13065 | 1719 | 41742520 | 13 | 77.25 |
| Terminal state | 7 | 13065 | 5087 | 41739152 | 4.39 | 18.33 |

| | | | | | | |
|---|---|---|---|---|---|---|
| Dizziness postural | 7 | 13065 | 6216 | 41738023 | 3.6 | 13.11 |
| Oxygen consumption increased | 7 | 13065 | 1833 | 41742406 | 12.2 | 71.67 |
| Oropharyngeal discomfort | 7 | 13065 | 5953 | 41738286 | 3.76 | 14.13 |
| Product supply issue | 7 | 13065 | 5042 | 41739197 | 4.43 | 18.59 |
| Intra-abdominal fluid collection | 7 | 13065 | 1649 | 41742590 | 13.56 | 81.07 |
| Therapy change | 6 | 13066 | 6737 | 41737502 | 2.84 | 7.17 |
| Endotracheal intubation | 6 | 13066 | 1752 | 41742487 | 10.94 | 53.98 |
| Cardiac flutter | 6 | 13066 | 4172 | 41740067 | 4.59 | 16.84 |
| Walking distance test abnormal | 6 | 13066 | 307 | 41743932 | 62.41 | 355.62 |
| Blood pressure systolic decreased | 6 | 13066 | 2750 | 41741489 | 6.97 | 30.6 |
| Pulmonary haemorrhage | 6 | 13066 | 4847 | 41739392 | 3.95 | 13.22 |
| Device power source issue | 6 | 13066 | 2264 | 41741975 | 8.46 | 39.39 |
| Postoperative wound infection | 6 | 13066 | 5327 | 41738912 | 3.6 | 11.24 |
| Infarction | 6 | 13066 | 4892 | 41739347 | 3.92 | 13.02 |
| Transplant | 6 | 13066 | 1644 | 41742595 | 11.65 | 58.23 |
| Product cleaning inadequate | 6 | 13066 | 2337 | 41741902 | 8.2 | 37.83 |
| Device operational issue | 6 | 13066 | 2935 | 41741304 | 6.53 | 28.03 |
| Hernia repair | 5 | 13067 | 1689 | 41742550 | 9.45 | 37.69 |
| Pulmonary pain | 5 | 13067 | 2254 | 41741985 | 7.08 | 26.07 |
| Urine output decreased | 5 | 13067 | 5188 | 41739051 | 3.08 | 7.01 |
| Pulseless electrical activity | 5 | 13067 | 3024 | 41741215 | 5.28 | 17.32 |
| Systemic scleroderma | 5 | 13067 | 576 | 41743663 | 27.72 | 127.68 |
| Pelvic fracture | 5 | 13067 | 5534 | 41738705 | 2.89 | 6.15 |
| Incorrect dosage administered | 5 | 13067 | 4025 | 41740214 | 3.97 | 11.08 |
| Left ventricular failure | 5 | 13067 | 2009 | 41742230 | 7.95 | 30.29 |
| Incorrect drug administration rate | 5 | 13067 | 2832 | 41741407 | 5.64 | 19.04 |
| Hysterectomy | 5 | 13067 | 5412 | 41738827 | 2.95 | 6.44 |
| Anuria | 5 | 13067 | 5100 | 41739139 | 3.13 | 7.24 |
| Tricuspid valve incompetence | 5 | 13067 | 3663 | 41740576 | 4.36 | 12.93 |
| Multiple allergies | 5 | 13067 | 5503 | 41738736 | 2.9 | 6.23 |
| Procedural complication | 5 | 13067 | 3781 | 41740458 | 4.22 | 12.28 |
| Oxygen therapy | 5 | 13067 | 1625 | 41742614 | 9.83 | 39.52 |
| Organ failure | 5 | 13067 | 1880 | 41742359 | 8.49 | 32.97 |
| Cardiac failure acute | 5 | 13067 | 4358 | 41739881 | 3.66 | 9.67 |

| | | | | | | |
|---|---|---|---|---|---|---|
| Rales | 5 | 13067 | 3681 | 41740558 | 4.34 | 12.82 |
| Portal hypertension | 5 | 13067 | 1824 | 41742415 | 8.75 | 34.25 |
| Procedural hypotension | 5 | 13067 | 465 | 41743774 | 34.34 | 160.11 |
| Intensive care | 5 | 13067 | 858 | 41743381 | 18.61 | 82.84 |
| Localised oedema | 4 | 13068 | 3055 | 41741184 | 4.18 | 9.67 |
| Idiopathic pulmonary fibrosis | 4 | 13068 | 2823 | 41741416 | 4.52 | 10.97 |
| Cardiac pacemaker replacement | 4 | 13068 | 345 | 41743894 | 37.03 | 138.6 |
| Sputum increased | 4 | 13068 | 1516 | 41742723 | 8.43 | 26.11 |
| Pulmonary arterial pressure abnormal | 4 | 13068 | 216 | 41744023 | 59.14 | 224.46 |
| Hypercapnia | 4 | 13068 | 1821 | 41742418 | 7.01 | 20.58 |
| Hypopnoea | 4 | 13068 | 2514 | 41741725 | 5.08 | 13.09 |
| Cardiac ablation | 4 | 13068 | 1246 | 41742993 | 10.25 | 33.29 |
| Biliary colic | 4 | 13068 | 1858 | 41742381 | 6.87 | 20.04 |
| Respiratory tract inflammation | 4 | 13068 | 348 | 41743891 | 36.71 | 137.35 |
| Renal function test abnormal | 4 | 13068 | 3118 | 41741121 | 4.1 | 9.35 |
| Paranasal sinus hypersecretion | 4 | 13068 | 2679 | 41741560 | 4.77 | 11.89 |
| Appendicectomy | 4 | 13068 | 2289 | 41741950 | 5.58 | 15.01 |
| Heart disease congenital | 4 | 13068 | 2401 | 41741838 | 5.32 | 14.01 |
| Hepatic neoplasm | 4 | 13068 | 2166 | 41742073 | 5.9 | 16.24 |
| Blood uric acid increased | 4 | 13068 | 3576 | 41740663 | 3.57 | 7.4 |
| Physical deconditioning | 4 | 13068 | 1410 | 41742829 | 9.06 | 28.6 |
| Cervical vertebral fracture | 3 | 13069 | 2356 | 41741883 | 4.07 | 6.93 |
| Red blood cell transfusion | 3 | 13069 | 1412 | 41742827 | 6.78 | 14.77 |
| Compression fracture | 3 | 13069 | 2792 | 41741447 | 3.43 | 5.16 |
| Sarcoidosis | 3 | 13069 | 2989 | 41741250 | 3.21 | 4.55 |
| Heart transplant | 3 | 13069 | 584 | 41743655 | 16.4 | 43.18 |
| Mechanical ventilation | 3 | 13069 | 1109 | 41743130 | 8.64 | 20.21 |
| Colostomy | 3 | 13069 | 1260 | 41742979 | 7.6 | 17.16 |
| Blood carbon monoxide increased | 3 | 13069 | 41 | 41744198 | 233.66 | 647.62 |
| Oesophageal pain | 3 | 13069 | 2333 | 41741906 | 4.11 | 7.04 |
| Appendix disorder | 3 | 13069 | 879 | 41743360 | 10.9 | 26.88 |
| Neonatal respiratory distress syndrome | 3 | 13069 | 2148 | 41742091 | 4.46 | 8.04 |
| Colonoscopy | 3 | 13069 | 1481 | 41742758 | 6.47 | 13.84 |
| Therapeutic product ineffective | 3 | 13069 | 3089 | 41741150 | 3.1 | 4.27 |

| | | | | | | |
|---|---|---|---|---|---|---|
| Transplant evaluation | 3 | 13069 | 447 | 41743792 | 21.43 | 58.05 |
| Peripheral arterial occlusive disease | 3 | 13069 | 2820 | 41741419 | 3.4 | 5.07 |
| Peripheral venous disease | 3 | 13069 | 2288 | 41741951 | 4.19 | 7.27 |
| Escherichia urinary tract infection | 3 | 13069 | 2745 | 41741494 | 3.49 | 5.32 |
| Skin neoplasm excision | 3 | 13069 | 518 | 41743721 | 18.49 | 49.36 |
| Pharyngeal haemorrhage | 3 | 13069 | 828 | 41743411 | 11.57 | 28.87 |
| Liver transplant | 3 | 13069 | 2040 | 41742199 | 4.7 | 8.71 |
| Oesophageal varices haemorrhage | 3 | 13069 | 1437 | 41742802 | 6.67 | 14.42 |
| Respiratory acidosis | 3 | 13069 | 2408 | 41741831 | 3.98 | 6.68 |
| Procedural haemorrhage | 3 | 13069 | 2850 | 41741389 | 3.36 | 4.97 |
| Aspiration pleural cavity | 3 | 13069 | 576 | 41743663 | 16.63 | 43.85 |
| Atrial septal defect repair | 3 | 13069 | 129 | 41744110 | 74.27 | 211.91 |
| Right ventricular enlargement | 3 | 13069 | 234 | 41744005 | 40.94 | 115.42 |
| Brain natriuretic peptide increased | 3 | 13069 | 1882 | 41742357 | 5.09 | 9.85 |
| Pulmonary hypertensive crisis | 3 | 13069 | 176 | 41744063 | 54.43 | 154.72 |
| Central venous catheterisation | 3 | 13069 | 2313 | 41741926 | 4.14 | 7.14 |
| Abdominal cavity drainage | 3 | 13069 | 321 | 41743918 | 29.84 | 82.86 |
| Oral mucosal erythema | 3 | 13069 | 1120 | 41743119 | 8.55 | 19.96 |
| Cardiac procedure complication | 3 | 13069 | 314 | 41743925 | 30.51 | 84.82 |
| Rhinovirus infection | 3 | 13069 | 2509 | 41741730 | 3.82 | 6.23 |
| Lip discolouration | 3 | 13069 | 797 | 41743442 | 12.02 | 30.2 |
| Heart and lung transplant | 3 | 13069 | 95 | 41744144 | 100.84 | 287.49 |
| Cardiac index abnormal | 3 | 13069 | 8 | 41744231 | 1197.53 | 2608.43 |
| Cardiac output decreased | 3 | 13069 | 514 | 41743725 | 18.64 | 49.79 |
| Traumatic fracture | 3 | 13069 | 488 | 41743751 | 19.63 | 52.72 |
